# Targeted high-level production of chuangxinmycin and its halogenated derivatives with antitubercular activity

**DOI:** 10.1186/s12934-025-02740-x

**Published:** 2025-05-19

**Authors:** Xiongfang Zhao, Yuan Lu, Xintong Zhang, Xiumin Zhang, Yu Du, Xingli Han, Yuting Zhu, Wei Yu, Linzhuan Wu, Xingxing Li, Yuanyuan Shi, Tianyu Zhang, Bin Hong

**Affiliations:** 1 CAMS Key Laboratory of Synthetic Biology for Drug Innovation, NHC Key Laboratory of Biotechnology for Microbial Drugs and State Key Laboratory of Bioactive Substances and Functions of Natural Medicines, Institute of Medicinal Biotechnology, Chinese Academy of Medical Sciences & Peking Union Medical College, Beijing, China; 2 State Key Laboratory of Respiratory Disease, Guangzhou Institutes of Biomedicine and Health, Chinese Academy of Sciences, Guangzhou, Guangdong China; 3 University of Chinese Academy of Sciences, Beijing, China; 4 School of Life Sciences, University of Science and Technology of China, Hefei, Anhui China; 5 Guangzhou National Laboratory, Guangzhou, Guangdong China

**Keywords:** *Mycobacterium tuberculosis*, Chuangxinmycin, High-level production, Halogenated derivatives

## Abstract

**Background:**

Chuangxinmycin (CM) is an old antibiotic from *Actinoplanes tsinanensis* CPCC, 200056, characterized by a dihydrothiopyrano[4,3,2-*cd*]indole scaffold and potent activity against *Mycobacterium tuberculosis.* Its congener norchuangxinmycin (NCM), which lacks antibacterial activity against various bacteria, unexpectedly retains antitubercular activity, indicating new mechanisms of action against *M. tuberculosis* in addition to tryptophan-tRNA synthetase inhibition. However, the variable low productivity and the limited number of active structural analogues represent a significant challenge for the future discovery and development of new anti-tuberculosis drugs involving CM and its derivatives.

**Results:**

Based on the elucidation of CM biosynthetic pathway, we employed a stepwise strategy by combining heterologous expression, activator overexpression, promoter optimization and fermentation media screening to achieve directed and high-level production of CM and its congener NCM. The highest yields achieved were 301 mg/L (a 20.1-fold increase) for CM and 117.6 mg/L (a 13.7-fold increase) for NCM. Furthermore, eleven halogenated CM derivatives were obtained through precursor-directed biosynthesis, with six of them being purified and structurally confirmed by HR-MS, HR-MS/MS and NMR. Bioactivity testing against *M. tuberculosis* H37Rv and clinical isolates of isoniazid/rifampin-resistant *M. tuberculosis* showed potent activity for 5-F-CM and 7-F-NCM.

**Conclusions:**

Synthetic biology techniques are well-suited for the targeted and high-level biosynthesis of CM and its derivatives. This study reports the highest laboratory-level yields of CM and NCM to date. This is the first instance of obtaining CM derivatives by biosynthesis rather than chemical synthesis, and it also marks the first report of halogenated NCM derivatives. High-level production of CM and its diverse analogues will provide a solid material foundation for advancing CM and its derivatives as potential anti-tuberculosis drug candidates.

**Supplementary Information:**

The online version contains supplementary material available at 10.1186/s12934-025-02740-x.

## Background

Mycobacterial infections have caused significant morbidity and mortality in humans [1], especially the pathogen *Mycobacterium tuberculosis* (Mtb), which has infected about a quarter of the global population to cause tuberculosis (TB) [1]. Remarkably, TB reclaimed its status as the world’s leading cause of death attributed to a single infectious agent in 2023, after a three-year hiatus prompted by the coronavirus disease (COVID-19) pandemic [1]. Alarmingly, TB caused 1.25 million deaths annually, nearly doubling the mortality rate of HIV/AIDS, alongside over 10 million new cases each year [1]. Although TB is treatable, multidrug-resistant TB remains a public health crisis [2]. Therefore, antibiotics that have novel drug targets and/or novel mechanisms of action are urgently needed.

In this context, chuangxinmycin (CM), a distinctive indole alkaloid antibiotic initially identified from the actinomycetes *Actinoplanes tsinanensis* CPCC 200056 in the 1970s [3], emerges as a promising candidate. Characterized by its unique dihydrothiopyrano[4,3,2-*cd*]indole structure, CM exhibits potent broad-spectrum anti-infective properties against both Gram-positive bacteria and Gram-negative bacteria in vitro. Its efficacy has been further demonstrated in vivo, showing activity in mouse models of *Escherichia coli* and *Shigella dysenteriae* infections, as well as in preliminary clinical trials [3]. CM is a naturally occurring inhibitor of bacterial tryptophan-tRNA synthetase (TrpRS) [4], a potential therapeutic target that has not yet been fully explored in clinical settings [5, 6]. Recently, this old antibiotic demonstrated its new fascination with significant activity against *M. tuberculosis* H37Rv and drug-resistant clinical isolates of *M. tuberculosis*, with a minimum inhibitory concentration (MIC = 0.78 ~ 1 µg/mL) [7]. Indolmycin, another TrpRS inhibitor, also demonstrated antitubercular activity with an MIC of approximately 3 µg/mL [8]. Additionally, CM’s congener norchuangxinmycin [9] (NCM, i.e., 3-demethylchuangxinmycin) showed similar activity against *M. tuberculosis* (MIC = 0.78 ~ 4 µg/mL) [7]. This finding attracted our attention enormously due to the potential of CM and its derivatives for the future discovery and development of new anti-TB drugs. Moreover, NCM and the recently discovered derivative 3-methylchuangxinmycin (MCM) displayed a marked reduction or even loss of antibacterial activity against most Gram-negative and Gram-positive bacteria such as *E. coli* and *S. aureus*, suggesting that the 3-methyl group in CM is pivotal for its antibacterial activity [7, 10]. In contrast, NCM retains a similar inhibitory activity against *M. tuberculosis* as CM [7], implying the involvement of an additional novel antimicrobial mechanism against *M. tuberculosis* [11]. This will offer new perspectives for the development novel anti-tuberculosis drugs.

**Fig. 1:**
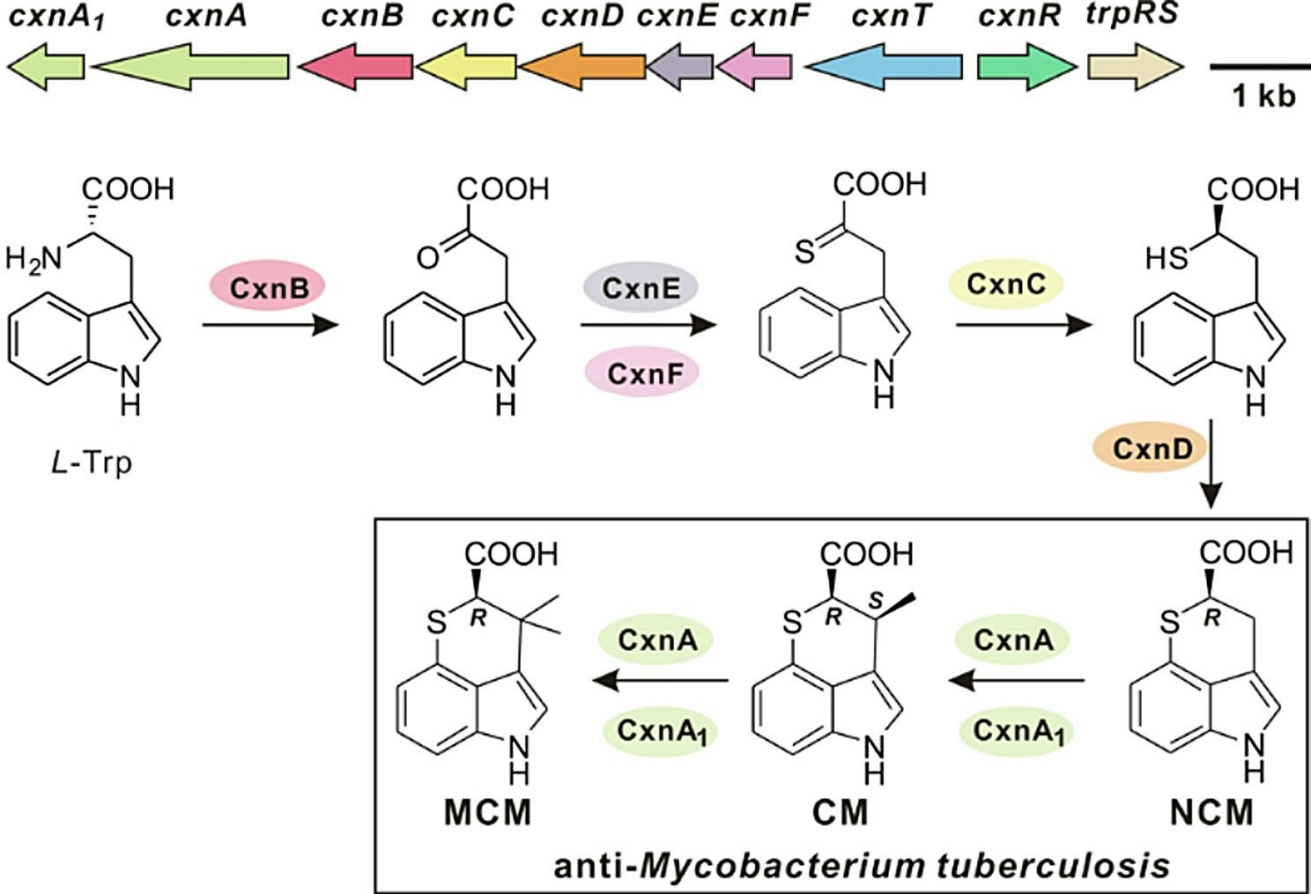
The biosynthetic gene cluster and pathway of CM

To enhance efficacy of CM for clinical applications, numerous CM derivatives have been chemically synthesized and evaluated over the past half century [4, 12–15]. However, the chemical synthesis of CM has encountered challenges in achieving stereochemical control over the two chiral carbons. Its antibacterial activity is highly dependent on its stereoselectivity, as only one of the four stereoisomers exhibits antibacterial activity [16]. The CM biosynthetic pathway in its producing strain *A. tsinanensis* has been elucidated recently [17–20] (Fig. 1). Through the identification of a potentially resistant gene, *trpRS*, the biosynthetic gene cluster *cxn* was identified and confirmed to be involved in CM production via heterologous expression (Table S1) [17, 18]. Starting with the processing of the tryptophan (Trp) precursor, transamination occurs through PLP-dependent aminotransferase CxnB to form an α-keto amino acid, which is then converted to an α-thione derivative for sulfur incorporation by the pathway-specific deubiquitinase-like sulfurtransferase CxnF and the ubiquitin-like sulfur carrier protein CxnE [20]. Following thione reduction by NAD(P)H-dependent reductase CxnC to yield a thiol intermediate, cytochrome P450 CxnD catalyzes C-S bond formation for intramolecular heterocyclization to achieve NCM [19]. Finally, C3-methylation occurs in a regio- and stereoselective manner to produce CM via the vitamin VB_12_-dependent radical SAM C-methyltransferase CxnA and CxnA_1_ [7, 21] (Fig. 1). Many known secondary metabolites derived from tryptophan, such as pyrrolnitrin [22], pyrroindomycin [23], rebeccamycin [24], and thienodolin [25, 26], can undergo halogenation reactions, as halogens usually affect the properties and activity of compounds. By leveraging the elucidated biosynthetic pathway of CM, various biosynthetic approaches can be employed for the directed production of CM at high levels and generating diverse CM analogues under precise stereochemical control.

In this study, we employed a stepwise strategy combining heterologous expression, activator overexpression, promoter selection, and optimization of fermentation media for directed and high-level production of CM and its congener NCM. Furthermore, eleven halogenated CM derivatives were obtained through precursor-directed biosynthesis, with six of them being purified and structurally confirmed by HR-MS, HR-MS/MS, and NMR. Bioactivity testing against *M. tuberculosis* H37Rv and clinical isolates of isoniazid/rifampin-resistant *M. tuberculosis* showed favorable activity for 5-F-CM, 7-F-NCM and 6-Cl-CM/NCM.

## Results

### Targeted high-level production of CM or NCM by heterologous expression

**Fig. 2:**
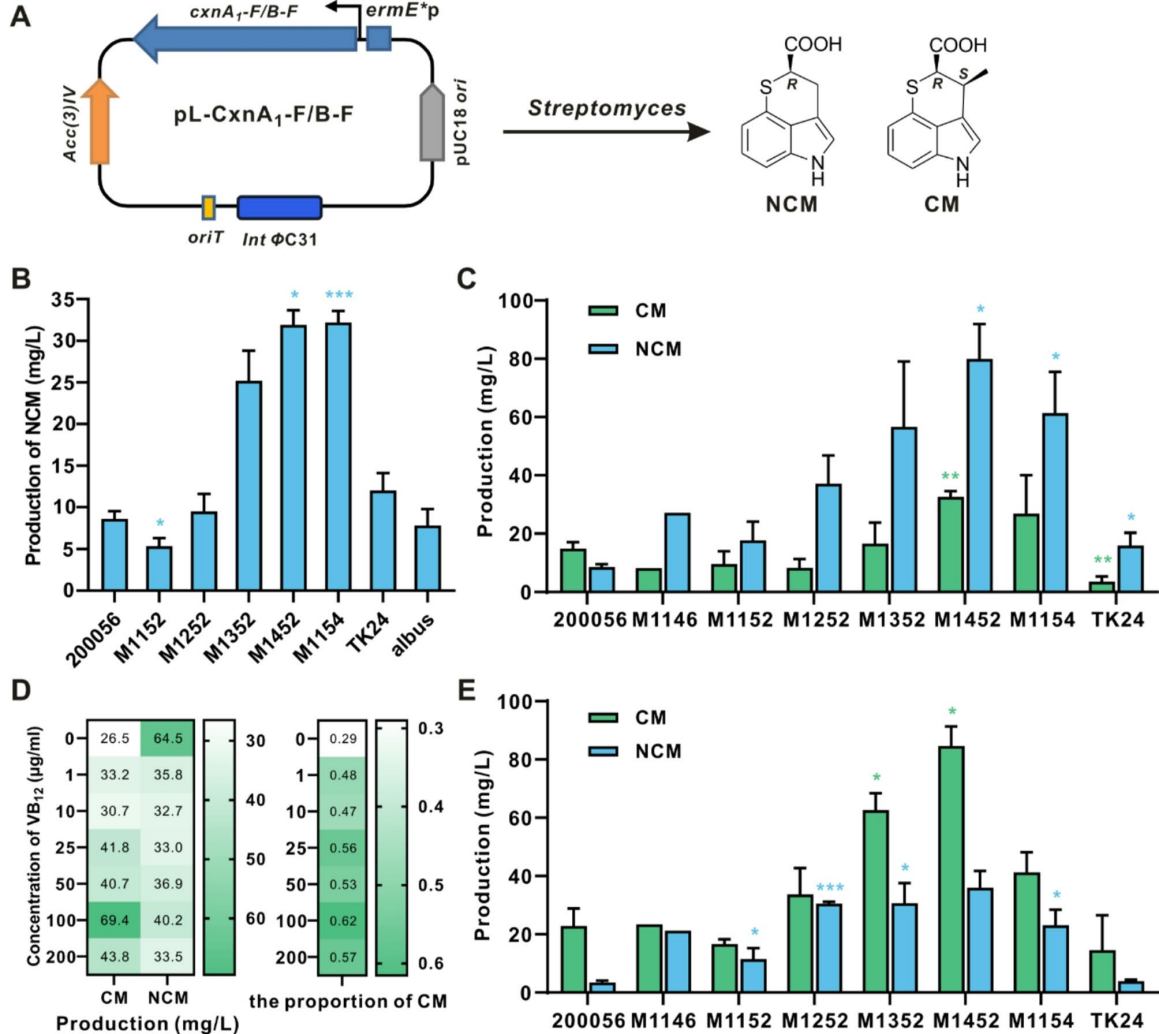
Heterologous production of CM and NCM in *Streptomyces*. (**A**) Schematic representation of constructions and heterologous expressions of pL-CxnA_1_-F and pL-CxnB-F. (**B**) Production of NCM in different *Streptomyces* hosts which possessed pL-CxnB-F when fermented on ISP2 plate. The yield of NCM in each strain was compared with that in the wild-type strain 200056 (Student’s *t* test, ^*^p < 0.05, ^***^p < 0.001). (**C**) Production of CM and NCM in different *Streptomyces* hosts which possessed pL-CxnA_1_-F when fermented on ISP2 plate. The yields of CM and NCM in each strain were compared with those in the wild-type strain 200056 (Student’s *t* test or Welch’s *t* test, ^*^p < 0.05, ^**^p < 0.01). (**D**) Optimization of VB_12_ concentration fed to fermentation medium ISP2 in *S. coelicolor* M1452/pL-CxnA_1_-F. (**E**) Production of CM and NCM in *Streptomyces*/pL-CxnA_1_-F with 100 µg/ml VB_12_ fed to ISP2 plate. The yields of CM and NCM in each strain were compared with those in the wild-type strain 200056 (Student’s *t* test or Welch’s *t* test, ^*^p < 0.05, ^***^p < 0.001). Values are presented as mean ± SEM (three independent conjugants for each strain)

To achieve targeted high-level production of CM and NCM through heterologous expression, we addressed the limitations of the original producing strain, *A. tsinanensis* CPCC 200056. This strain exhibits low and unstable CM production, averaging 15.0 mg/L, and the presence of CM congeners such as NCM and MCM complicates downstream separation and purification processes. Notably, NCM, which is a minor component with an average yield of 8.6 mg/L, is challenging to produce using this strain. Therefore, we pursued heterologous expression as a strategy for directed and high-level production of CM and NCM, respectively. The biosynthetic genes for CM and NCM were cloned into an expression vector, resulting in the plasmids pL-CxnA_1_-F and pL-CxnB-F, respectively (Fig. 2A). Then they were individually introduced into different *Streptomyces* hosts for expression, including *S. coelicolor* strains M1146, M1152, M1154 [27], and M1252/M1352/M1452 [28] (which allow multi-copy chromosomal integration), as well as *S. lividans* TK24 and *S. albus*.

The fermentation results showed that NCM was produced in all the heterologous hosts, with *S. coelicolor* M1452/pL-CxnB-F and M1154/pL-CxnB-F exhibiting the highest yield of NCM at 32.0 mg/L, a 3.7-fold increase compared to *A. tsinanensis* CPCC 200056 when fermented on ISP2 plates (Fig. 2B). In the initial fermentation of *Streptomyces* strains expressing CxnA_1_-F, the maximum yield of both CM and NCM was achieved with *S. coelicolor* M1452/pL-CxnA_1_-F at approximately 122.5 mg/L. However, under these conditions, NCM was the main product at the highest production of 88.4 mg/L, which seems more suitable for NCM production. The proportion of CM was only about 30% (Fig. 2C), which may be due to the low efficiency of methyltransferase in these heterologous hosts. As CxnA encodes a vitamin B_12_-dependent radical SAM C-methyltransferase [7, 21], the CM/NCM ratio increased significantly when 1 µg/ml VB_12_ was fed to fermentation medium. Optimization of VB_12_ concentration improved the CM proportion to 62% at the concentration of 100 µg/ml in *S. coelicolor* M1452/pL-CxnA_1_-F (Fig. 2D). Then 100 µg/ml VB_12_ was fed to the fermentation of different *Streptomyces* strains expressing CxnA_1_-F, the CM production was significantly enhanced to various levels in all expression strains. The maximal combined production of CM and NCM reached 120.6 mg/L in *S. coelicolor* M1452/pL-CxnA_1_-F, similar to the levels without VB_12_ addition. As expected, the highest yield of CM reached 84.6 mg/L, which is 5.6 times higher than that in *A. tsinanensis* CPCC 200056 (Fig. 2E).

### Optimization of promoters of cluster-situated regulator to improve CM production

**Fig. 3:**
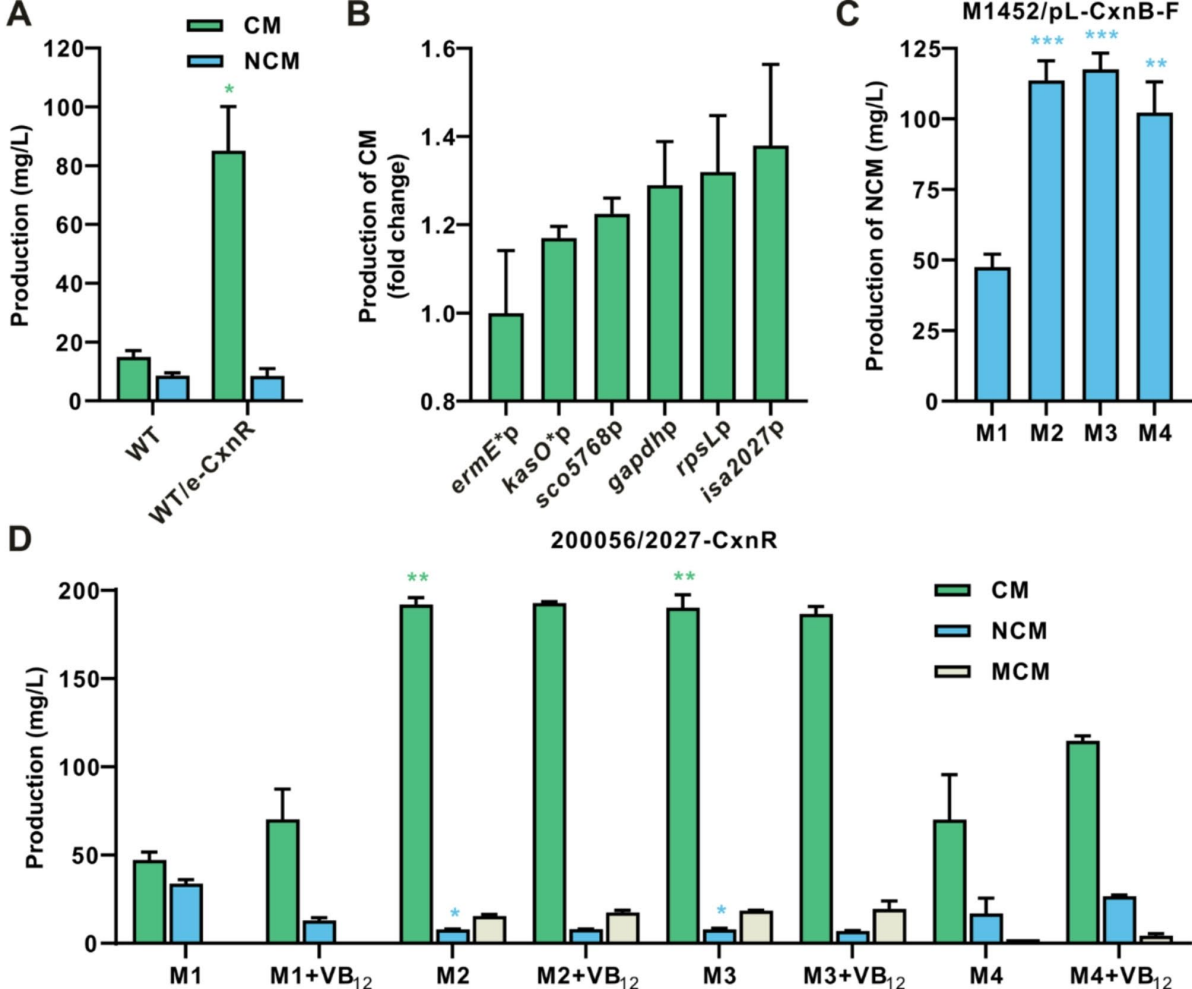
Effect of different promoters and fermentation media on the production of CM and NCM. (**A**) Analysis of CM and NCM production when *cxnR* overexpressed in wild-type strain and fermented on ISP2 plate. WT: CM-producing strain *A. tsinanensis* CPCC 200056; WT/e-CxnR: *cxnR* overexpression in 200056. The yield of CM in the two strains was compared with each other (Student’s *t* test, ^*^p < 0.05). (**B**) Effect of different promoters driving the expression of *cxnR* on CM production in *A. tsinanensis* CPCC 200056. Different promoters represented different strains. ermE^*^p: 200056/e-CxnR; kasO^*^p: 200056/k-CxnR; *sco5768*p: 200056/5768-CxnR; *gapdh*p: 200056/g-CxnR; *rpsL*p: 200056/r-CxnR; *isa2027*p: 200056/2027-CxnR. (**C**) NCM production in *S. coelicolor* M1452/pL-CxnB-F cultivated in different fermentation media. The yield of NCM in each medium was compared with that in M1 medium (Student’s *t* test, ^**^p < 0.01, ^***^p < 0.001). (**D**) Production of CM and its congeners in 200056/2027-CxnR cultivated in different fermentation media. The yields of CM and NCM in each medium were compared with those in M1 medium (Welch’s *t* test, ^*^p < 0.05, ^**^p < 0.01). Values are presented as mean ± SEM (three independent conjugants for each strain)

In the original producing strain *A. tsinanensis* CPCC 200056, the proportion of CM was 60% and further increased to about 80% by VB_12_ supply on ISP2 plate (Figure S1). The proportion of CM in heterologous expression strain was much lower (Fig. 2C), and adding VB_12_ to the fermentation broth could only increase the proportion of CM to about 70% (Fig. 2D). Therefore, heterologous expression in *S. coelicolor* M1452 may be not suitable for CM though its yield increased. Within the CM biosynthetic gene cluster, *cxnR* encodes the only cluster-situated positive regulator [17]. In the original producing strain, overexpression of *cxnR* under the control of a strong constitutive promoter ermE^*^p (from *S. erythraeus*) significantly improved the production of CM. The production level of CM reached 85.1 mg/L, equivalent to that of *S. coelicolor* M1452/pL-CxnA_1_-F (Fig. 3A). What’s more, the CM/NCM ratio increased to 9:1, while no MCM was detected in 200056/e-CxnR. In this case, *cxnR* overexpression in the wild-type CM-producing strain is more suitable for stereoselective and efficient production of CM. To further enhance the expression level of the *cxnR* gene in *A. tsinanensis* CPCC 200056, five different promoters were used to substitute ermE^*^p in the overexpression plasmid. These included four well-known constitutive promoters, namely, *gapdh*p (from *Eggerthella lenta*) [29], *rpsL*p (from *Xylanimonas cellulosilytica*) [29], kasO^*^p (from *S. coelicolor*) [30], and *sco5768*p (from *S. coelicolor*) [31], as well as one promoter of the RNA polymerase sigma factor in *Streptomyces* sp. CPCC 204095, *isa2027*p [32]. Under the same fermentation conditions on ISP2 plates, the five promoters showed superior performance compared to ermE^*^p, with 200056/2027-CxnR exhibiting the largest increase in CM production by 1.4 times (Fig. 3B).

To achieve the highest level of customized CM or NCM production, we optimized the composition of culture media. We screened 4 fermentation media with or without VB_12_ (100 µg/ml) and expanded the fermentation from 6 cm to 15 cm diameter plates to achieve a more stable and higher yield. The selected culture media included ISP2 (M1), fish powder fermentation medium (M2) [7], corn steep liquor fermentation medium (M3), and cottonseed cake powder fermentation medium (M4), each containing different carbon and nitrogen sources. HPLC analysis revealed that NCM production of M1452/pL-CxnB-F was significantly increased when cultured in M2, M3 and M4 media, reaching a maximum of 117.6 mg/L in M3 medium (Fig. 3C), which is 13.7 times that of the original producing strain and the highest yield reported in the literature so far. The engineered strain 200056/2027-CxnR cultivated in M2 and M3 showed significantly higher CM production with low levels of NCM and MCM, even without VB_12_ supplementation (Fig. 3D). The maximal CM production reached 192.4 mg/L when 200056/2027-CxnR was cultivated in M2 medium. These results showed that optimization of the fermentation conditions significantly increased CM and NCM production in the engineered strains. Medium selection for M1452/pL-CxnA_1_-F also showed a significant increase in total CM and NCM production in M3 medium (Figure S2). However, the highest proportion of CM only reached 70% even with VB_12_ supplementation. This suggested that the efficiency of methyltransferase for CM production in heterologous expression strain *S. coelicolor* M1452 is lower than that in the original strain, making the original CM-producing strain is more suitable for CM expression. To further evaluate the productivity of the engineered strain 200056/2027-CxnR in the optimized media, scale-up fermentation was carried out in a 42 L fermenter containing 20 L liquid fermentation medium M2. The yield of CM reached 301 mg/L, 20.1 times the production of original producing strain, with 19 mg/L for MCM (less than 10% of the total). This represents the highest titer of CM reported to date at the laboratory level to date, facilitating the investigation of mechanisms necessary for developing CM as a lead compound against tuberculosis.

### Production of halogenated CM derivatives by precursor-directed biosynthesis

**Fig. 4:**
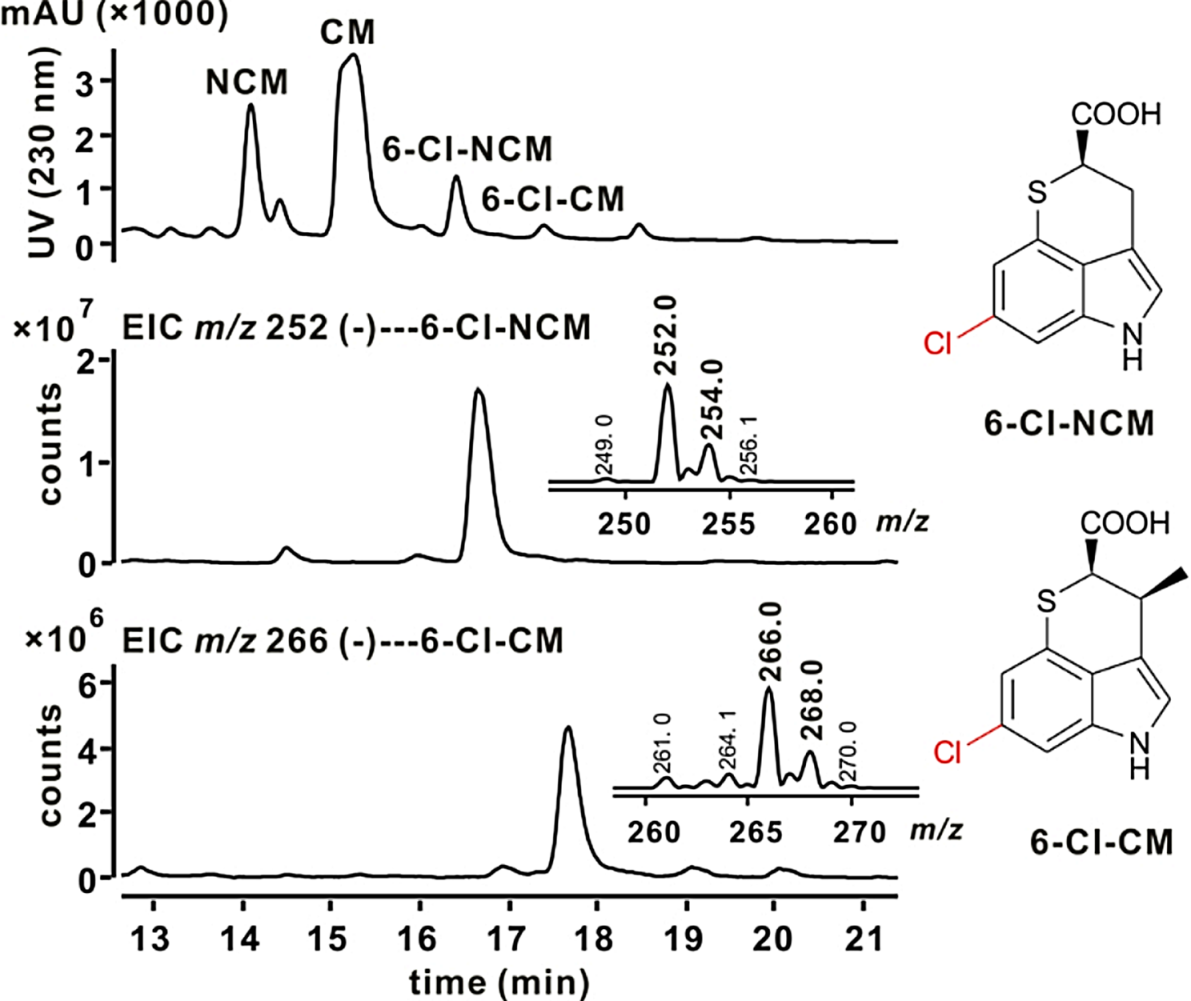
Production of chlorinated metabolites by feeding 6-Cl-Trp

**Fig. 5:**
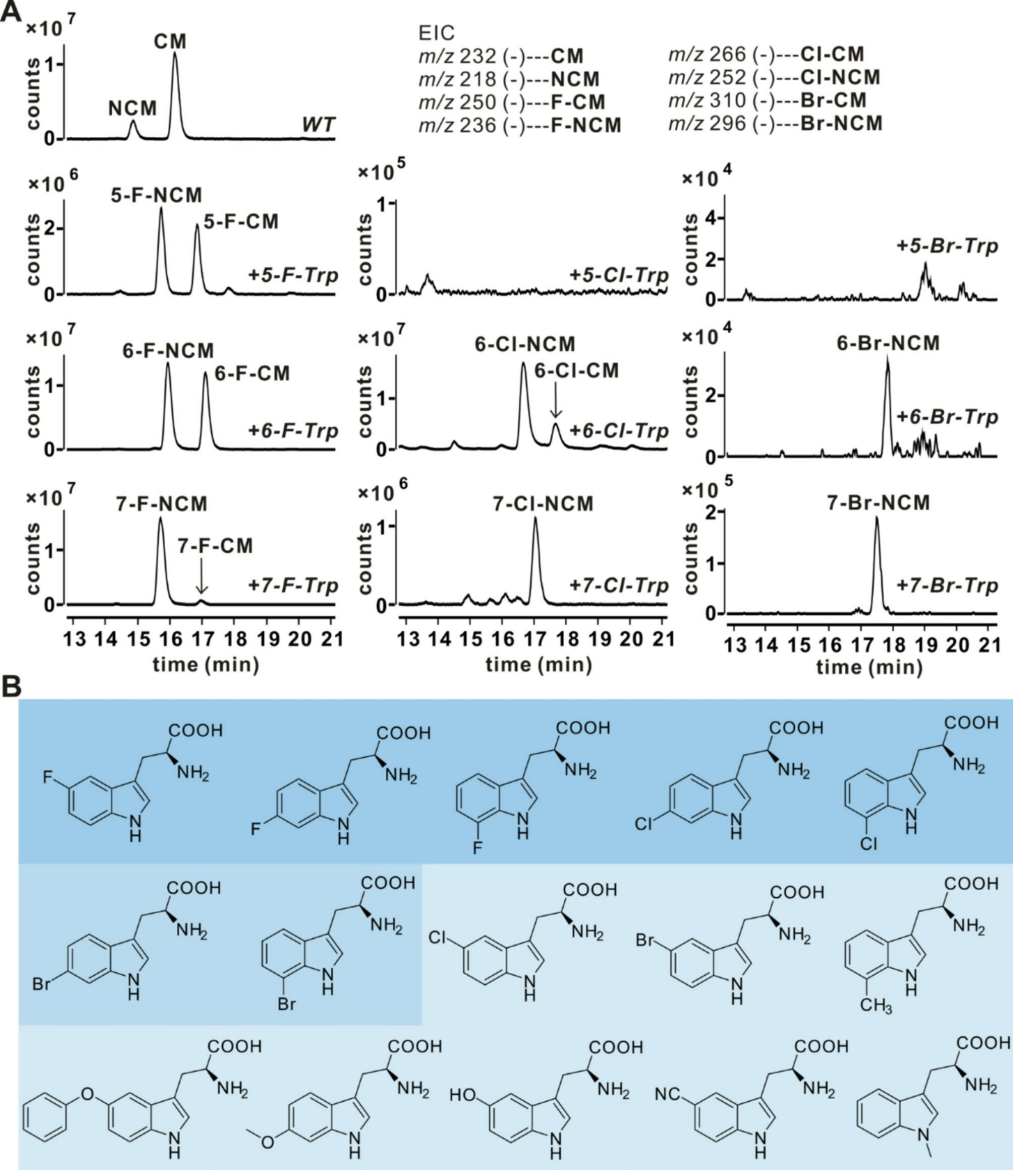
Addition of tryptophan derivatives to the biosynthetic platform allows incorporation of substituted tryptophans into CM/NCM. (**A**) LC-MS analysis of metabolites of 200056 when fed 5/6/7-F/Cl/Br-Trp. Corresponding extracted ion chromatogram (EIC) was shown on the top. (**B**) Tryptophan derivatives tested. Dark blue shows tryptophan derivatives that were incorporated into an analogue of NCM (> 38% of underivatized CM by LC-MS analysis; Table S3) and further purified; medium blue indicates tryptophan derivatives that were incorporated into an analogue of NCM at lower levels (1–12% of underivatized CM by LC-MS analysis; Table S3) but were not further verified by purification; and light blue indicates tryptophan derivatives that did not show detectable incorporation into NCM

To produce halogenated CM derivatives, we introduced tryptophan 6-halogenase (ThdHI) [25] into CM-producing strains, including the original strain *A. tsinanensis* CPCC 200056 and the heterologous expression strain *S. coelicolor* M1452/pL-CxnA_1_-F. However, no halogenated CM derivatives were detected. Subsequently, we directly fed 6-Cl-Trp to the producing strains to generate CM/NCM derivatives. LC-MS analysis showed the production of chlorinated metabolites, 6-Cl-NCM (*m/z* [M-H]^−^ 252/254) and 6-Cl-CM (*m/z* [M-H]^−^ 266/268), in both fermentation broths (Fig. 4). The yield of chlorinated derivatives in M1452/pL-CxnA_1_-F was lower than in 200056. Then we optimized the feeding concentration of 6-Cl-Trp in original producing strain, finding that the yield of 6-Cl-NCM and 6-Cl-CM showed little change with feeding concentration (Table S2). Therefore, we selected 0.5 mM as the lowest feeding concentration for 6-Cl-Trp considering the economic cost. To determine the scope of tryptophan derivatives accepted by our biosynthetic pathway, we fed a variety of tryptophan derivatives, including halogenated tryptophans with different halogen atoms at different positions (i.e. 5/6/7-F/Cl/Br-Trp) and other substituents at different positions (i.e. 1/5/6/7-CH_3_/OCH_3_/CN/OH/BnO-Trp), to *A. tsinanensis* CPCC 200056 and monitored the production of new derivatives by LC-MS. Among the halogenated tryptophans tested, fluorinated tryptophans substituted at the 5-, 6- and 7-positions and chlorinated tryptophans substituted at the 6- and 7-positions were the best accepted by the biosynthetic pathway (Fig. 5 and Table S3). The incorporations became more and more difficult as the size of the substituents increased, especially for 5-halogenated tryptophans, as 5-Cl-Trp and 5-Br-Trp failed to produce derivatives. Brominated tryptophans substituted at the 6- and 7-positions could incorporate and produce small amounts of corresponding NCM-derivatives. Moreover, the production of NCM halogenated derivatives was higher than CM halogenated derivatives (Table S4), possibly due to the specificity of methyltransferase (CxnA/A_1_). Other tested tryptophan derivatives, such as 5-hydroxy/cyano/ benzyloxy, 6-methoxy, 7-methyl and 1-methyl-L-tryptophan, were not incorporated into the biosynthetic pathway to produce derivatives (Fig. 5 and Table S3), perhaps because these larger substituents may have steric hindrance in some of the enzyme active sites, or the substrate specificity of the downstream biosynthetic enzymes limited the activation of analogues, reducing the efficiency of turnover. Overall, cultures fed with fluorinated tryptophans produced a higher amount of CM/NCM derivatives than those fed with chlorinated tryptophans (Table S4). This is the first time CM derivatives have been obtained by biosynthesis for the stereoselective and customizable production. A total of eleven halogenated derivatives were obtained, marking the first report of halogenated NCM derivatives.

**Fig. 6:**
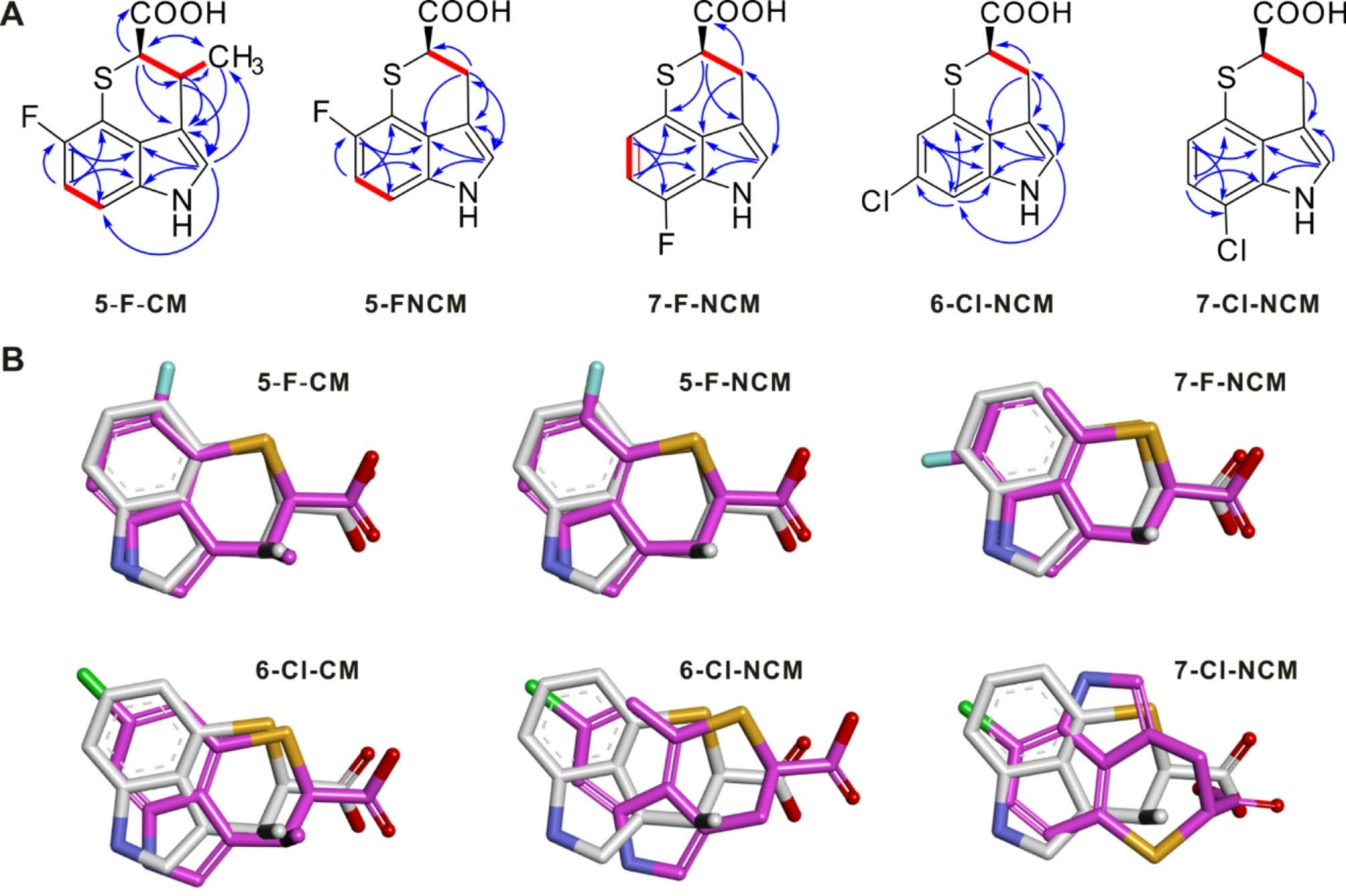
Structure confirmation of new halogenated derivatives and their molecular docking into the target TrpRS. (**A**) Key HMBC (blue arrows) and ^1^H -^1^H COSY (red thick bonds) correlations of 5-F-CM, 5-F-NCM, 7-F-NCM, 6-Cl-NCM and 7-Cl-NCM. (**B**) Docking of chuangxinmycin derivatives made in this study into a chuangxinmycin-bound crystal structure of TrpRS from *Bacillus stearothermophilus* (PDB: 7CKI). Docked molecules (magenta) are compared with active site-bound chuangxinmycin (white) from the crystal structure

To characterize these halogenated derivatives, we scaled the fermentations to obtain sufficient quantities for chemical structure confirmation and activity assessment. The target compounds in the fermentation broth were extracted with EtOAc, concentrated to get crude extract, then isolated by silica gel column chromatography and purified by semi-preparative HPLC. As a result, six new CM analogues were obtained and designated as 5-F-CM, 5-F-NCM, 7-F-NCM, 6-Cl-CM, 6-Cl-NCM, 7-Cl-NCM, respectively. Their structures were elucidated by electrospray ionization high-resolution mass spectrometry (ESI-HRMS), ESI-HRMS/MS and nuclear magnetic resonance (NMR) spectroscopic analysis, except 6-Cl-CM, which could not be further confirmed by NMR due to the limited quantity (Fig. 6A, Table S5-S6 and Figure S3-S37).

We then tested six derivatives together with CM against *M. tuberculosis*, as well as *M. phlei*, *M. smegmatis* and some other bacteria. The MIC values demonstrated that 5-F-CM and 7-F-NCM showed the most potent inhibition of *M. tuberculosis* H37Rv compared to any of the other derivatives, comparable to CM/NCM, with 5-F-NCM and 7-Cl-NCM completely lost the activity (Table 1). Furthermore, both CM and 5-F-CM showed strong activity against clinical isolates of isoniazid/rifampin-resistant *M. tuberculosis*. The reduced bioactivity of the chlorinated compounds may be due to the bulky chlorinated substituent hindering their ability to bind to the TrpRS target, consistent with docking studies of the derivatives into a bacterial TrpRS structure (Fig. 6B). Additionally, CM showed no or weak inhibition activity against the other tested bacteria, except *M. smegmatis* (Table 1 and Table S7), indicating its specific antitubercular activity. What’s more, CM and its analogues exhibited no obvious cytotoxicity, with CC_50_ values ≥ 64 µg/mL at least, across multiple cell lines such as Huh7, Huh7.5, and Vero cells (Table S7).

**Table 1:** Activities of chuangxinmycin derivatives

Compounds	MIC (µg/mL)					
	*M. tuberculosis*				*M. phlei*	*M. smegmatis*
	H37Rv	M9483	M6600	M3551		
CM	1	2	1	2	1	4
NCM	4	/	/	/	16	> 32
5-F-CM	4	4	4	8	16	> 32
5-F-NCM	> 32	/	/	/	> 32	> 32
7-F-NCM	4	/	/	/	/	> 32
6-Cl-CM	16	/	/	/	32	> 32
6-Cl-NCM	32	64	32	64	> 32	> 32
7-Cl-NCM	> 32	/	/	/	> 32	> 32
INH	0.0625	R	R	R	/	/
RIF	0.125	R	R	R	/	/
LZD	0.25	S	S	S	/	/
STR	/	/	/	/	8	4

Note: *M. tuberculosis* H37Rv, standard strain; M6600, M3551, and M9483 are clinically isolated multidrug-resistant strains of *M. tuberculosis*, resistant to INH, at 0.1 µg/mL and RIF, at 1.0 µg/mL, sensitive to LZD, at 2.0 µg/mL. INH, isoniazid; RIF, rifampicin; LZD, linezolid; STR: streptomycin. /, not detected

## Discussion

Antibiotic-resistant bacteria pose a great threat to human health, and the discovery and clinical approval of new antibiotic have sharply declined since the 1980s. The ‘rediscovery’ of known but underdeveloped antibiotics represented a new strategy to enhance antibiotic development [33]. Chuangxinmycin is an example of such an antibiotic, originally identified in 1964 for its broad-spectrum antibacterial activity, targeting clinically undeveloped TrpRS. The recent discovery of chuangxinmycin’s activity against *M. tuberculosis* has reignited our interest in elucidating its antitubercular mechanism. NCM with similar antitubercular activity but reduced effectiveness against most Gram-positive and negative bacteria, suggests that the dihydrothiopyrano[4,3,2-*cd*]indole scaffold may inhibit *M. tuberculosis* through unique mechanisms beyond TrpRS inhibition [11]. However, the low titer of chuangxinmycin in its native producer remains a critical limitation. Therefore, generating an over-producing strain is crucial for the scale-up production. Cloning the biosynthetic gene cluster and heterologous expression in a suitable host are common methods to boost secondary metabolite production.

In our study, the total yield of CM and its congener NCM in *S. coelicolor* M1452/pL-CxnA_1_-F increased fivefold compared to the native producer. Optimization of VB_12_ supplementation increased the proportion of CM to 70%, indicating insufficient methylation efficiency in *S. coelicolor* M1452. Overexpression of a cluster-situated positive regulator CxnR significantly increased CM production in the native producing strain, achieving a CM proportion of over 90% in various fermentation conditions. CxnR is a typical LysR-type transcriptional regulator, featuring an N-terminal DNA-binding domain followed by a long helix that connects to a C-terminal effector-binding domain. A deeper understanding of its role in controlling CM biosynthesis would strengthen the findings here, and could effectively and strategically guide efforts to further enhance CM production. Then the selection of five constitutive promoters for *cxnR* overexpression increased CM production by 1.4 times compared to ermE^*^p. Among these, the *isa2027* promoter performed best in *A. tsinanensis* CPCC 200056, outperforming strong promoters like ermE^*^p, kasO^*^p and *rpsL*p. Notably, *isa2027*p encodes a principal sigma-like transcriptional factor in *Streptomyces* sp. CPCC 204095, responsible for housekeeping gene transcription, and could be a potent promoter in other actinomycetes species. Optimizing the culture medium further improved CM production, increasing it by 12.8-fold in M2 medium and 20.1-fold in a 42 L fermenter. Similarly, NCM production increased by 13.7-fold with no congeners when *S. coelicolor* M1452/pL-CxnB-F was cultivated in M3 medium. In summary, high-level production of CM and NCM was achieved through a combinatorial strategy.

Motivated by these advancements, a series of halogenated derivatives were obtained by precursor-directed feeding. Introduction of some tailoring genes, especially tryptophan 6-halogenase (ThdHI) [25], unexpectedly failed to produce any new analogues (data not shown). By contrast, the high-yield producing strain may incorporate halogenated substituted indoles at 5, 6 and 7-position into the CM and NCM, facilitating and overcoming the challenges of producing stereoselective compounds with one or two chiral carbons in NCM and CM, respectively. Compared to the traditional chemical synthesis for producing CM and its derivatives, the biosynthetic approach not only simplifies the complicated synthesis route and enhances environmental compatibility with a green and efficient process, but also yields CM with specific stereoconfiguration that possesses antibacterial activity. A lot of CM derivatives have been chemically synthesized and evaluated over the last 25 years. These structural modifications mainly focused on carboxyl, methyl, and indole nitrogen atom of CM and the structure–activity relationships of CM inferred that only sterically smaller analogues afforded significant inhibition [4, 16]. However, a CM prodrug with a carboxyl benzoyloxy methyl ester showed better in vitro antibacterial activities on Gram-positive bacteria including *Mycobacterium tuberculosis* H37Rv, which was reported in a literature [15] and a Chinese patent [34]. Moreover, the prodrug derivatives of indole ring substituted by monobromide and tribromide showed comparable activity against *M. tuberculosis* H37Rv [34]. Therefore, brominated derivatives were also taken into our consideration when exploring the possibility of producing derivatives through biosynthetic approaches. Regrettably, brominated derivatives were produced at a too low level to be further verified in this study. However, chlorinated derivatives showed inferior antibacterial activity compared to fluorinated derivatives with smaller sizes. Combined with the docking results, it is inferred again that only sterically smaller analogues exhibit significant inhibition activity. The production of halogenated derivatives primarily depends on the substrate acceptance of biosynthetic enzymes, which could be further improved through strain and enzyme engineering. Although most derivatives showed weak antitubercular activity, fluorinated derivatives of CM (5-F-CM) and NCM (7-F-NCM) showed potent bioactivity against *M. tuberculosis*, indicating their potential as useful molecules for development of new antibiotics. Further structure modifications of CM might enhance antitubercular activity, similar to the situation of indolmycin, another known TrpRS inhibitor. For example, the 4’, 6’-difluorine-substituted indolmycin showed higher antitubercular activity than the 4’-fluorine-substituted indolmycin [8]. Moreover, the installed fluorine provides a selectively functionalizable handle for synthetic modification for further structure diversification. Overall, this work lays a foundation for stereoselective and customizable generation of CM and its derivatives, highlighting the value of synthetic biology in targeted high-level production and diversification of CM.

## Conclusion

A stepwise strategy was employed, encompassing heterologous expression, activator overexpression, promoter selection and optimization of fermentation media, to achieve directed and high-level production of CM and NCM. The maximal production of CM reached 301 mg/L, a 20.1-fold increase, while that of NCM reached 117.6 mg/L, representing the highest yield reported at laboratory level to date. Furthermore, eleven halogenated CM derivatives were synthesized through precursor-directed biosynthesis, with six of them purified and structurally confirmed. Notably, 5-F-CM and 7-F-NCM showed potent activity against *M. tuberculosis.* The high-level production of CM and its diverse analogues provides a material foundation for studying the antitubercular mechanism of CM and the development of CM or its derivatives as potential anti-TB drug candidates.

## Materials and methods

### Strains and culture conditions

The chuangxinmycin**-**producing strain *Actinoplanes tsinanensis* CPCC 200056, was grown at 28 °C on solid ISP2 medium (yeast extract 4 g/L, malt extract 10 g/L, glucose 4 g/L, agar 20 g/L) for sporulation and fermentation. Three medium 65 plate (4 g of glucose, 4 g of yeast extract, 10 g of malt extract, 2 g of CaCO_3_, 12 g of agar, 1 L, pH 7.2) [35] was used for conjugation between *A. tsinanensis* and *Escherichia coli*. The heterologous expression host strain *Streptomyces coelicolor* M1146, M1152, M1154 [27], M1252/M1352/M1452 [28] (multi-copy chromosomal integration), *S. lividans* TK24, *S. albus* and their derivatives were cultured at 28 °C on Mannitol soya flour (MS) agar [36], which was also used for conjugation between *Streptomyces* and *Escherichia coli*. ISP2 medium was used for fermentation of *Streptomyces*, and liquid phage medium [37] was used to culture *Streptomyces* for genomic DNA isolation. *E. coli* DH5α [38] was used as a host for general cloning experiments. *E*. *coli* ET12567/pUZ8002 [39] was used for conjugal transfer according to established protocol. All *E. coli* strains were incubated in Luria-Bertani medium (LB) at 37 °C. Apramycin (Am, 50 µg/mL), kanamycin (Km, 50 µg/mL), chloramphenicol (Cm, 30 µg/mL), hygromycin B (Hyg, 200 µg/mL) and nalidixic acid (25 µg/mL) were used for selection of recombinant *E. coli* or *Streptomyces* strains. Strains, plasmids, and primers used in this study are listed in Table S8 and S9.

### Fermentation and analysis of chuangxinmycin and its derivatives

*A. tsinanensis* CPCC 200056, *S. coelicolor*, *S. lividans* TK24, *S. albus* and their derivatives were fermented on 6 cm diameter ISP2 plate at 28 °C for 7 days. Every culture on ISP2 plate was cut into squares (about 1 cm × 1 cm in size), and extracted once with 2-fold ethyl acetate overnight. The ethyl acetate was concentrated to the same volume (250 ~ 500 µL) and filtered with organic membrane filter of 0.22 μm for HPLC-MS analysis which was carried out by HPLC coupled to an ESI mass spectrometer (1100–6410 Triple Quad LC/MS (Agilent, America)). HPLC analysis was performed on a ZORBAX SB-C8 column (4.6 × 250 mm, 5 μm, Agilent, America) at a flow rate of 0.8 mL/min with detection wavelength of 230 nm and column temperature of 25 °C. Gradient elution was carried out with MeCN-H_2_O (water phase containing 0.1% glacial acetic acid): 0–30 min, 15 − 100%, MeCN gradient elution. Chuangxinmycin’s standard curve was drawn for quantitative analysis by peak area at 230 nm at different concentrations.

### Construction of the engineered strains for high-level production of CM and NCM

The plasmid pL-CxnA_1_-F was constructed by seamless assembly using NEBuilder HiFi DNA Assembly Master Mix (NEB), which was same as the construction of pL-CxnB-F [19]. Restriction enzyme digested vector and PCR generated inserts were assembled with varied overlaps (20–40 bp). First, CxnA_1_ABCDEF was divided into 3 fragments, which were respectively amplified by PCR. Then, they were cloned into the *Nde*I-*Bam*HI sites of pL646 [40], a pSET152 [41]-derived expression plasmid with a strong constitutive promoter ermE^*^p in the upstream of the multiple cloning sites, to get plasmid pL-CxnA_1_-F. Finally, pL-CxnA_1_-F and pL-CxnB-F were introduced into *E. coli* ET12567/pUZ8002 respectively and then transferred into *Streptomyces* by conjugation for heterologous expression.

For construction of cluster-situated positive regulator overexpression strains with different promoters driving the expression of *cxnR*, the coding region of *cxnR* was PCR-amplified from *A. tsinanensis* CPCC 200056 using the primers CxnR-F and CxnR-R and then cut with *Spe*I and *Eco*RI. It was ligated into the constructed pSET152-based expression plasmids with different promoters including kasO^*^p, *sco5768*p, *gapdh*p, *rpsL*p and *isa2027*p to generate pSET-k-CxnR, pSET-5768-CxnR, pSET-g-CxnR, pSET-r-CxnR and pSET-2027-CxnR, respectively. All of the derived plasmids were confirmed by restriction enzyme digestion analysis. Then these plasmids and pL-CxnR [17] were transformed into *E. coli* ET12567/pUZ8002 and conjugated with *A. tsinanensis* CPCC 200056 to generate 200056/e-CxnR, 200056/k-CxnR, 200056/5768-CxnR, 200056/g-CxnR, 200056/r-CxnR, and 200056/2027-CxnR, respectively.

Four fermentation media were screened for chuangxinmycin and 3-demethylchuangxinmycin production. Compositions of these four culture media were as follows: Medium M1 (ISP2), M2 [7] (starch 50 g/L, soybean cake meal 15 g/L, fish meal 5 g/L, dipotassium hydrogen phosphate 1 g/L, sodium thiosulfate 5 g/L, cobalt chloride 1 mg/L, calcium carbonate 10 g/L, agar 15 g/L), M3 (starch 50 g/L, soybean cake meal 15 g/L, corn steep liquor 5 g/L, dipotassium hydrogen phosphate 1 g/L, sodium thiosulfate 5 g/L, cobalt chloride 1 mg/L, calcium carbonate 10 g/L, agar 15 g/L), and M4 (starch 50 g/L, cottonseed cake powder 25 g/L, yeast extract 5 g/L, dipotassium hydrogen phosphate 1 g/L, sodium thiosulfate 5 g/L, cobalt chloride 1 mg/L, calcium carbonate 10 g/L, agar 15 g/L). Fresh spore suspension was spread on a diameter of 15 cm dish containing 50 ml fermentation medium and incubated at 28 °C for 7 days. Chuangxinmycin and 3-demethylchuangxinmycin production level in these derivatives were determined by HPLC-MS as described above.

Batch experiments were performed in a 42 L stirred-tank bioreactor (Biostat C30 plus, Germany) equipped with pH, temperature and pO2 probes. Cultivation of 200056/2027-CxnR proceeded in a 100 mL volume of liquid fermentation medium M2 at 28 °C under agitation at 220 rpm. After 2 days of culture, a 5% seed culture was transferred into the bioreactor containing 20 L liquid fermentation media M2. The aeration was kept at 0.5 vvm (air/culture volume/min) and the agitation rate was set around 250 rpm. The culture was cultivated at 28 °C for 100 h before fermentation broth were collected for chuangxinmycin analysis.

### Purification and characterization of chuangxinmycin derivatives

Fresh spore suspension of *A. tsinanensis* CPCC 200056 was spread on a diameter of 15 cm dish containing 50 ml fermentation medium with 0.2 mM 5-F-Trp and incubated at 28 °C for 7 days. The agar cultures were pooled (ca. 5 L) and extracted twice with two-fold volume of ethyl acetate for 48 h. The organic layers were collected and vacuum-dried to afford 3.2 g crude extract. Then it was fractionated by normal-phase silica gel column flash chromatography on Büchi C-850 (Germany), eluting with a linear gradient of 0–10% solvent B (A: 0.1% [v/v] HOAc in CH_2_Cl_2_; B: 0.1% [v/v] HOAc in CH_3_OH) to give fractions containing CM and its derivatives. These fractions were combined and evaporated under reduced pressure to get a yellowish residue (600 mg). Subsequently, a reverse-phase C18 flash chromatography was applied for preliminary separation of each compound on Büchi C-850 using gradient elution of 10–100% solvent B (A: 0.1% [v/v] HOAc in H_2_O; B: 0.1% [v/v] HOAc in CH_3_OH) to afford a preparation containing 5-F-CM and 5-F-NCM (47 mg). Then the preparation was applied on semi-preparative HPLC equipped with an LC-20AT pump and DAD detector (Shimadzu, Japan) using a Xselect CSH C18 OBD prep column (10 × 250 mm, 5 μm, Waters, USA) for purification, using an isocratic gradient of MeCN-H_2_O (25: 75, water phase containing 0.1% HOAc). Finally, 5-F-CM (2.6 mg) and 5-F-NCM (0.9 mg) were obtained.

The fermentation and isolation of other halogenated derivatives from feeding 6-F-Trp/7-F-Trp/6-Cl-Trp/7-Cl-Trp into fermentation medium at a final concentration of 0.2 mM are nearly the same as described above for 5-F-CM and 5-F-NCM. The isocratic gradient of MeCN-H_2_O was adjusted during semi-preparation. Finally, a series of halogenated derivatives were obtained, including 7-F-NCM (2.7 mg), 6-Cl-CM (0.33 mg), 6-Cl-NCM (1.53 mg) and 7-Cl-NCM (1.93 mg). 6-F-CM and 6-F-NCM were not successfully purified and gained for the poor fermentation status resulting in low yields. These new compounds were structurally identified by ESI-HRMS, ESI-HRMS/MS and NMR, except 6-Cl-CM.

### Antibacterial assay

To determine the inhibition activity of chuangxinmycin halogenated derivatives against *M. tuberculosis* H37Rv and clinically isolated strains, a measurement was conducted by the microplate Alamar blue assay as reported previously [7]. Briefly, Fresh culture suspensions of *M. tuberculosis* H37Rv or clinical isolates were diluted with 7H9 medium to reach 10^6^ CFU/mL and then distributed into 96-well plates (100 µL/well). The CM and its derivatives were dissolved in DMSO and then serially diluted with 7H9 medium to final concentrations ranging from 64 µg/mL to 0.125 µg/mL when added into the above 96-well plates (4 µL/well). MIC is defined as the lowest concentration of samples that prevents the conversion of the blue color into pink color. Triplicate wells per drug concentration were used. Isoniazid (INH), rifampicin (RIF) and linezolid (LZD) were used as controls.

The microdilution assay recommended by the Clinical and Laboratory Standards Institute [42] was employed to determine the MICs of the other bacterial strains as described previously [43]. Strains were cultured in Mueller-Hinton broth (MHB) [44] and the final bacterial suspension was adjusted to 10^6^ cells/ml in MHB medium. Dilutions of test compounds were substituted with MHB medium as described above. Then, triplicate 100 µL transfers of serial dilutions were placed into 96-well plates, after which 100 µL of bacterial suspension was added to each well. After incubation for 10 h at 37 °C, the MIC was defined as the lowest concentration of samples that inhibited the growth of the test organism detected visually. Streptomycin served as a positive control.

### Molecular docking

Molecular docking experiment to test derivatives binding to a TrpRS was conducted by using the CDOCKER program from Discovery Studio (DS) software (version 2019, Biovea Inc., Omaha, NE, USA) as previously described [7]. Ligand molecules were prepared and energy was minimized using CHARMm force field in DS. The TrpRS model used for docking was from *Geobacillus stearothermophilus* (PDB: 7CKI) [45] and prepared by the “Prepare protein” option equipped in the DS using the CHARMm force field. The co-crystallized ligand chuangxinmycin and water molecules were removed from the model before docking. Active site cavity was defined around the inbound ligand as a sphere with radius as 5.283Å. The other parameters were set at their default values. The best pose demonstrating the highest -CDOCKER energy was chosen from the 10 conformations for further study.

### Cytotoxicity assay

The cytotoxic effects of compounds were measured on various cells, including human hepatocellular carcinoma Huh7 and Huh7.5 cells as well as African green monkey kidney Vero E6 cells. All the test compounds, with an initial concentration of 128 µg/mL or 64 µg/mL, were serially diluted threefold for seven dilutions and co-incubated with cells for 48 h respectively. The cell morphology was monitored by Olympus microscope and the 50% cytotoxic concentration (CC_50_) was determined by the Reed-Muench method [46].

### Statistical analysis

All data for the productions of chuangxinmycin and its analogues were presented as mean ± SEM. Statistical analyses involving comparison between groups of data were performed with Student’s *t* test (two-tailed) or Welch’s *t* test (two-tailed) as applicable by GraphPad Prism 9.5. Differences were considered statistically significant at ^*^p < 0.05, ^**^p < 0.01, and ^***^p < 0.001.

## Electronic supplementary material

Below is the link to the electronic supplementary material.

Supplementary Material 1

## Data Availability

No datasets were generated or analysed during the current study.
