# Supplementary material for: Targeted high-level production of chuangxinmycin and its halogenated derivatives with antitubercular activity
Source: Microb Cell Fact. 2025 May 19;24:113. doi: 10.1186/s12934-025-02740-x (PMC12087186; doi:10.1186/s12934-025-02740-x)
Supplement: Supplementary file 1 — Supplementary Material 1 [file 12934_2025_2740_MOESM1_ESM.docx]

**Targeted high-level production of chuangxinmycin and its halogenated derivatives with antitubercular activity**

Xiongfang Zhao^1^, Yuan Lu^1^, Xintong Zhang^1^, Xiumin Zhang^1^, Yu Du^1^, Xingli Han^2,3^, Yuting Zhu^2,4^, Wei Yu^2,5^, Linzhuan Wu^1^, Xingxing Li^1^, Yuanyuan Shi^1*^, Tianyu Zhang^2,3,5*^, Bin Hong^1*^

^1^CAMS Key Laboratory of Synthetic Biology for Drug Innovation, NHC Key Laboratory of Biotechnology for Microbial Drugs and State Key Laboratory of Bioactive Substances and Functions of Natural Medicines, Institute of Medicinal Biotechnology, Chinese Academy of Medical Sciences & Peking Union Medical College, Beijing, China

^2^State Key Laboratory of Respiratory Disease, Guangzhou Institutes of Biomedicine and Health, Chinese Academy of Sciences, Guangzhou, Guangdong, China.

^3^University of Chinese Academy of Sciences, Beijing, China.

^4^School of Life Sciences, University of Science and Technology of China, Hefei, Anhui, China.

^5^Guangzhou National Laboratory, Guangzhou, Guangdong, China.

^*^Corresponding authors: Bin Hong, Tianyu Zhang, Yuanyuan Shi

Email: Bin Hong, [binhong69@hotmail.com](mailto:binhong69@hotmail.com), [hongbin@imb.pumc.edu.cn;](mailto:hongbin@imb.pumc.edu.cn;) Tianyu Zhang, [zhang_tianyu@gibh.ac.cn;](mailto:zhang_tianyu@gibh.ac.cn;) Yuanyuan Shi, [luckshiyuanyuan@163.com](mailto:luckshiyuanyuan@163.com)

**Table of contents**

[Table S1. Chuangxinmycin biosynthetic gene cluster and gene functions 4](#_Toc193372480)

[Table S2. Production of derivatives 6-Cl-NCM/6-Cl-CM under different feeding concentrations of 6-Cl-Trp 5](#_Toc193372481)

[Table S3. Substrate scope analysis. 5](#_Toc193372482)

[Table S4. Production of halogenated derivatives 6](#_Toc193372483)

[Table S5. NMR data for 5-F-CM, 5-F-NCM and 7-F-NCM 7](#_Toc193372484)

[Table S6. NMR data for 6-Cl-NCM and 7-Cl-NCM 8](#_Toc193372485)

[Table S7. Activities and cytotoxicity of chuangxinmycin derivatives 9](#_Toc193372486)

[Table S8. Bacterial strains and plasmids used in this study 10](#_Toc193372487)

[Table S9. Primers used in this study 13](#_Toc193372488)

[Figure S1. The change of the proportion of CM with 100 μg/ml VB_12_ fed to ISP2 plate in *Streptomyces*/pL-CxnA_1_-F and 200056. 14](#_Toc193372489)

[Figure S2. Production of CM and NCM in *S. coelicolor* M1452/pL-CxnA_1_-F cultivated in different fermentation media. 15](#_Toc193372490)

[Figure S3. (-)-HR-ESIMS spectrum of 5-F-CM 16](#_Toc193372491)

[Figure S4. (-)-HR-ESIMS/MS spectrum of 5-F-CM 17](#_Toc193372492)

[Figure S5. ^1^H NMR spectrum of 5-F-CM in CD_3_COCD_3_ 18](#_Toc193372493)

[Figure S6. ^13^C NMR spectrum of 5-F-CM in CD_3_COCD_3_ 19](#_Toc193372494)

[Figure S7. HSQC spectrum of 5-F-CM in CD_3_COCD_3_ 20](#_Toc193372495)

[Figure S8. HMBC spectrum of 5-F-CM in CD_3_COCD_3_ 21](#_Toc193372496)

[Figure S9. ^1^H-^1^H COSY spectrum of 5-F-CM in CD_3_COCD_3_ 22](#_Toc193372497)

[Figure S10. (-)-HR-ESIMS spectrum of 5-F-NCM 23](#_Toc193372498)

[Figure S11. (-)-HR-ESIMS/MS spectrum of 5-F-NCM 24](#_Toc193372499)

[Figure S12. ^1^H NMR spectrum of 5-F-NCM in CD_3_OD 25](#_Toc193372500)

[Figure S13. ^13^C NMR spectrum of 5-F-NCM in CD_3_OD 26](#_Toc193372501)

[Figure S14. HSQC spectrum of 5-F-NCM in CD_3_OD 27](#_Toc193372502)

[Figure S15. HMBC spectrum of 5-F-NCM in CD_3_OD 28](#_Toc193372503)

[Figure S16. ^1^H-^1^H COSY spectrum of 5-F-NCM in CD_3_OD 29](#_Toc193372504)

[Figure S17. ^1^H NMR spectrum of 7-F-NCM in CD_3_OD 30](#_Toc193372505)

[Figure S18. ^13^C NMR spectrum of 7-F-NCM in CD_3_OD 31](#_Toc193372506)

[Figure S19. HSQC spectrum of 7-F-NCM in CD_3_OD 32](#_Toc193372507)

[Figure S20. HMBC spectrum of 7-F-NCM in CD_3_OD 33](#_Toc193372508)

[Figure S21. ^1^H-^1^H COSY spectrum of 7-F-NCM in CD_3_OD 34](#_Toc193372509)

[Figure S22. (-)-HR-ESIMS spectrum of 6-Cl-CM 35](#_Toc193372510)

[Figure S23. (-)-HR-ESIMS/MS spectrum of 6-Cl-CM 36](#_Toc193372511)

[Figure S24. (-)-HR-ESIMS spectrum of 6-Cl-NCM 37](#_Toc193372512)

[Figure S25. (-)-HR-ESIMS/MS spectrum of 6-Cl-NCM 38](#_Toc193372513)

[Figure S26. ^1^H NMR spectrum of 6-Cl-NCM in CD_3_OD 39](#_Toc193372514)

[Figure S27. ^13^C NMR spectrum of 6-Cl-NCM in CD_3_OD 40](#_Toc193372515)

[Figure S28. HSQC spectrum of 6-Cl-NCM in CD_3_OD 41](#_Toc193372516)

[Figure S29. HMBC spectrum of 6-Cl-NCM in CD_3_OD 42](#_Toc193372517)

[Figure S30. ^1^H-^1^H COSY spectrum of 6-Cl-NCM in CD_3_OD 43](#_Toc193372518)

[Figure S31. (-)-HR-ESIMS spectrum of 7-Cl-NCM 44](#_Toc193372519)

[Figure S32. (-)-HR-ESIMS/MS spectrum of 7-Cl-NCM 45](#_Toc193372520)

[Figure S33. ^1^H NMR spectrum of 7-Cl-NCM in CD_3_OD 46](#_Toc193372521)

[Figure S34. ^13^C NMR spectrum of 7-Cl-NCM in CD_3_OD 47](#_Toc193372522)

[Figure S35. HSQC spectrum of 7-Cl-NCM in CD_3_OD 48](#_Toc193372523)

[Figure S36. HMBC spectrum of 7-Cl-NCM in CD_3_OD 49](#_Toc193372524)

[Figure S37. ^1^H-^1^H COSY spectrum of 7-Cl-NCM in CD_3_OD 50](#_Toc193372525)

[References 51](#_Toc193372526)

# Table S1. Chuangxinmycin biosynthetic gene cluster and gene functions

| Gene | Size  (aa) | Protein homologue | Identity/  Similarity  (100%) | Function |
| --- | --- | --- | --- | --- |
| *cxnA_1_* | 240 | hypothetical protein (WP_086718244.1), *Streptomyces angustmyceticus* | 81/85 | an unknown DUF5825 family protein, a partner protein to cxnA, methylation[1] |
| *cxnA* | 623 | maturation radical SAM protein 1 (WP_063754376.1 RiPP), *Streptomyces* sp. NRRL S-1813 | 93/96 | VB_12_-dependent radical SAM protein, methylation[1] |
| *cxnB* | 362 | ThnJ, aminotransferase (AMR44310.1), *Streptomyces* sp. FXJ1.172 | 52/63 | PLP-dependent aminotransferase |
| *cxnC* | 318 | 2-dehydropantoate 2-reductase (WP_030988196.1), *Streptomyces* sp. NRRL S-1813 | 87/90 | NAD(P)H-dependent reductase |
| *cxnD* | 404 | ThnC, P450 (AMR44303.1), *Streptomyces* sp. FXJ1.172 | 37/48 | cytochrome P450[2] |
| *cxnE* | 100 | MoaD family protein (KRT67113.1), Candidatus Dadabacteria CSP1-2 | 43/71 | sulfur carrier protein |
| *cxnF* | 238 | ThnF, hypothetical protein (AMR44306.1), *Streptomyces* sp. FXJ1.172 | 44/61 | deubiquitinase-like sulfurtransferase[3] |
| *cxnT* | 496 | MFS transporter (WP_051818417.1), *Streptomyces* sp. NRRL S-1813 | 86/90 | transporter |
| *cxnR* | 313 | LysR family transcriptional regulator (AJK58337.1), *Amycolatopsis lurida* NRRL 2430 | 37/50 | regulator |
| *trpRS* | 300 | Ind0, tryptophanyl-tRNA synthetase (AJT38681.1), *Streptomyces griseus* subsp. *griseus* | 73/83 | tryptophan-tRNA synthetase |

# Table S2. Production of derivatives 6-Cl-NCM/6-Cl-CM under different feeding concentrations of 6-Cl-Trp

| Concentration of 6-Cl-Trp | derivatives | Production (mg/L) |
| --- | --- | --- |
| 0.5 mM | 6-Cl-NCM | 2.0 |
|  | 6-Cl-CM | 0.6 |
| 1 mM | 6-Cl-NCM | 2.0 |
|  | 6-Cl-CM | 0.6 |
| 2 mM | 6-Cl-NCM | 2.2 |
|  | 6-Cl-CM | 1.0 |

Table S3. Substrate scope analysis. Approximate amounts of derivatives of NCM/CM are given as a percentage of underivatized CM detected in the same sample from LC-MS extracted ion chromatograms at the corresponding *m/z* for each compound.

| tryptophan fed to 200056 | NCM-derivative (***m/z***) | % of CM | CM-derivative (***m/z***) | % of CM |
| --- | --- | --- | --- | --- |
| 5-fluoro-tryptophan | 5-F-NCM (238) | 71 | 5-F-CM (250) | 31 |
| 6-fluoro-tryptophan | 6-F-NCM (238) | 153 | 6-F-CM (250) | 114 |
| 7-fluoro-tryptophan | 7-F-NCM (238) | 153 | 7-F-CM (250) | 5 |
| 5-chloro-tryptophan | 5-Cl-NCM (252/254) | 0 | 5-Cl-CM (266/268) | 0 |
| 6-chloro-tryptophan | 6-Cl-NCM (252/254) | 53 | 6-Cl-CM (266/268) | 10 |
| 7-chloro-tryptophan | 7-Cl-NCM (252/254) | 38 | 7-Cl-CM (266/268) | 0 |
| 5-bromo-tryptophan | 5-Br-NCM (296/298) | 0 | 5-Br-CM (310/312) | 0 |
| 6-bromo-tryptophan | 6-Br-NCM (296/298) | 1 | 6-Br-CM (310/312) | 0 |
| 7-bromo-tryptophan | 7-Br-NCM (296/298) | 12 | 7-Br-CM (310/312) | 0 |
| 5-benzyloxy-tryptophan | 5-BnO-NCM (324) | 0 | 5-BnO-CM (338) | 0 |
| 5-hydroxy-tryptophan | 5-OH-NCM (234) | 0 | 5-OH-CM (248) | 0 |
| 5-cyano-tryptophan | 5-CN-NCM (243) | 0 | 5-CN-CM (257) | 0 |
| 6-methoxy-tryptophan | 6-MeO-NCM (248) | 0 | 6-MeO-CM (262) | 0 |
| 7-methyl- tryptophan | 7-Me-NCM (232) | 0 | 7-Me-CM (246) | 0 |
| 1-methyl-tryptophan | 1-Me-NCM (232) | 0 | 1-Me-CM (246) | 0 |

# Table S4. Production of halogenated derivatives

| tryptophan fed to 200056 | Production of NCM-derivative (mg/L) | Production of CM-derivative (mg/L) |
| --- | --- | --- |
| 5-fluoro-tryptophan | 1.2 (5-F-NCM) | 0.6 (5-F-CM) |
| 6-fluoro-tryptophan | 6.1 (6-F-NCM) | 5.0 (6-F-CM) |
| 7-fluoro-tryptophan | 9.0 (7-F-NCM) | / |
| 6-chloro-tryptophan | 2.0 (6-Cl-NCM) | 0.6 (6-Cl-CM) |
| 7-chloro-tryptophan | 2.3 (7-Cl-NCM) | / |
| 7-bromo-tryptophan | 1.1 (7-Br-NCM) | / |

# Table S5. NMR data for 5-F-CM, 5-F-NCM and 7-F-NCM

|  | **5-F-CM** | | **5-F-NCM** | | **7-F-NCM** | |
| --- | --- | --- | --- | --- | --- | --- |
| Position | *δ*_C_ | *δ*_H_ (*J* in Hz) | *δ*_C_ | *δ*_H_ (*J* in Hz) | *δ*_C_ | *δ*_H_ (*J* in Hz) |
| 1 | 171.5 | - | - | - | 175.6 | - |
| 2 | 52.0 | 4.3, s | 48.7 | 4.03, d (10.04) | 46.6 | 4.10, dd (8.8, 4.7) |
| 3 | 31.5 | 3.80, s | 28.6 | 3.48, dd (15.19, 3.17) | 27.8 | 3.42, dd (15.5, 3.9) |
|  |  |  |  | 3.16, dd (15.31, 10.05) |  | 3.24, dd (15.5, 8.9) |
| 4 | 117.5 | - | 120.0 | - | 111.9 | - |
| 5 | 122.0 | 7.26, s | 122.5 | 7.04, s | 122.2 | 7.06, s |
| 6(NH) | - | - | - | - | - | - |
| 7 | 131.7 | - | 132.2 | - | 123.7 | - |
| 8 | 109.1 | 7.12, dd (8.67, 3.58) | 108.9 | 7.01, dd (8.69, 3.53) | 121.2 | - |
| 9 | 111.1 | 6.88, t (8.67) | 111.1 | 6.81, dd (10.20, 8.69) | 108.2 | 6.76, dd (11.6, 7.9) |
| 10 | 151.9 | - | 152.4 | - | 115.7 | 6.70, dd (7.9, 3.6) |
| 11 | 110.3 | - | 111.9 | - | 148.3 | - |
| 12 | 124.4 | - | 125.1 | - | 127.7 | - |
| 13(S) | - | - | - | - | - | - |
| 14 | 18.9 | 1.32, d (6.65) | - | - | - | - |

^1^H and ^13^C NMR spectra data (*δ*) were obtained at 600 MHz for 5-F-CM in CD_3_COCD_3_ on Bruker-600 spectrometer, 5-F-NCM in CD_3_OD on Bruker-600 spectrometer, 7-F-NCM in CD_3_OD on a VNS-600 spectrometer at 20 °C

# Table S6. NMR data for 6-Cl-NCM and 7-Cl-NCM

|  | **6-Cl-NCM** | | **7-Cl-NCM** | |
| --- | --- | --- | --- | --- |
| Position | *δ*_C_ | *δ*_H_ (*J* in Hz) | *δ*_C_ | *δ*_H_ (*J* in Hz) |
| 1 |  |  |  |  |
| 2 | 47.8 | 4.13, m | 50.7 | 4.02, d(15.04) |
| 3 | 28.3 | 3.47, m | 29.0 | 3.51, d(15.04) |
|  |  | 3.23, dd(15.40, 9.22) |  | 3.17, dd(15.28, 10.51) |
| 4 | 111.4 | - | 113.4 | - |
| 5 | 121.6 | 7.02, s | 121.6 | 7.06, s |
| 6(NH) | - | - | - | - |
| 7 | 135.6 | - | 132.6 | - |
| 8 | 115.6 | 6.78, s | 113.7 | - |
| 9 | 128.6 | - | 122.6 | 7.00, d(7.76) |
| 10 | 108.9 | 7.09, s | 116.4 | 6.75, d(7.76) |
| 11 | 129.0 | - | 127.4 | - |
| 12 | 123.1 | - | 126.1 | - |
| 13(S) | - | - | - | - |

^1^H and ^13^C NMR spectra data (*δ*) were obtained at 600 MHz for 6-Cl-NCM and 7-Cl-NCM in CD_3_COCD_3_ on Bruker-600 spectrometer at 20 °C

# Table S7. Activities and cytotoxicity of chuangxinmycin derivatives

| Compounds | MIC (µg/mL) | | | | CC_50_ (µg/mL) | | |
| --- | --- | --- | --- | --- | --- | --- | --- |
|  | *B. subtilis* | *S. aureus* | *E. coli ΔtolC* | *P. aeruginosa 11* | Huh7 | Huh7.5 | Vero |
| CM | 16 | 8 | 16 | >32 | >128 | >128 | 64 |
| NCM | 16 | >32 | 32 | / | >128 | >128 | / |
| 5-F-CM | >32 | >32 | >32 | >32 | >64 | / | >64 |
| 5-F-NCM | >32 | >32 | >32 | >32 | >64 | / | >64 |
| 7-F-NCM | / | >32 | / | / | / | / | / |
| 6-Cl-CM | >32 | >32 | >32 | >32 | 64 | / | / |
| 6-Cl-NCM | >32 | / | >32 | >32 | >64 | / | / |
| 7-Cl-NCM | / | >32 | >32 | >32 | >64 | / | / |
| STR | 2 | 8 | 2 | 8 | / | / | / |

STR: streptomycin; /, not detected.

# Table S8. Bacterial strains and plasmids used in this study

| **Strains/plasmids** | **Relevant characteristics** | **Reference** |
| --- | --- | --- |
| **Strains** |  |  |
| *Actinoplanes tsinanensis* |  |  |
| CPCC 200056 | Wild-type strain (chuangxinmycin-producing strain) | [4] |
| 200056/e-CxnR | *A. tsinanensis* CPCC 200056 with the plasmid pL-CxnR, Am^r^ | [5] |
| 200056/k-CxnR | *A. tsinanensis* CPCC 200056 with the plasmid pSET-k-CxnR, Am^r^ | This study |
| 200056/5768-CxnR | *A. tsinanensis* CPCC 200056 with the plasmid pSET-5768-CxnR, Am^r^ | This study |
| 200056/g-CxnR | *A. tsinanensis* CPCC 200056 with the plasmid pSET-g-CxnR, Am^r^ | This study |
| 200056/r-CxnR | *A. tsinanensis* CPCC 200056 with the plasmid pSET-r-CxnR, Am^r^ | This study |
| 200056/2027-CxnR | *A. tsinanensis* CPCC 200056 with the plasmid pSET-2027-CxnR, Am^r^ | This study |
| *Streptomyces coelicolor* |  |  |
| M1146 | The heterologous expression host strain, *Δact, Δred, Δcpk, Δcda* | [6] |
| M1152 | The heterologous expression host strain, *Δact, Δred, Δcpk, Δcda, rpoB(C1298T)* | [6] |
| M1252 | M1152 carrying one artificial ΦC31 *attB* site located at the CPK BGC | [7] |
| M1352 | M1152 carrying two artificial ΦC31 *attB* sites located at the CPK and RED BGCs, respectively | [7] |
| M1452 | M1152 carrying three artificial ΦC31 *attB* sites located at the CPK, RED and CDA BGCs, respectively | [7] |
| M1154 | The heterologous expression host strain, *Δact, Δred, Δcpk, Δcda, rpoB(C1298T), rpsL(A262G)* | [6] |
| M1152/pL-CxnB-F | M1152 carrying one-copy integration of plasmid pL-CxnB-F | This study |
| M1252/pL-CxnB-F | M1252 carrying two-copy integration of plasmid pL-CxnB-F | This study |
| M1352/pL-CxnB-F | M1352 carrying three-copy integration of plasmid pL-CxnB-F | This study |
| M1452/pL-CxnB-F | M1452 carrying four-copy integration of plasmid pL-CxnB-F | This study |
| M1154/pL-CxnB-F | M1154 carrying one-copy integration of plasmid pL-CxnB-F | This study |
| M1146/pL-CxnA_1_-F | M1146 carrying one-copy integration of plasmid pL-CxnA_1_-F | This study |
| M1152/pL-CxnA_1_-F | M1152 carrying one-copy integration of plasmid pL-CxnA_1_-F | This study |
| M1252/pL-CxnA_1_-F | M1252 carrying two-copy integration of plasmid pL-CxnA_1_-F | This study |
| M1352/pL-CxnA_1_-F | M1352 carrying three-copy integration of plasmid pL-CxnA_1_-F | This study |
| M1452/pL-CxnA_1_-F | M1452 carrying four-copy integration of plasmid pL-CxnA_1_-F | This study |
| M1154/pL-CxnA_1_-F | M1154 carrying one-copy integration of plasmid pL-CxnA_1_-F | This study |
| *Streptomyces lividans* TK24 | The heterologous expression host strain, a plasmid-free derivative of *S. lividans* 66 | [8] |
| TK24/pL-CxnB-F | TK24 carrying one-copy integration of plasmid pL-CxnB-F | This study |
| TK24/pL-CxnA_1_-F | TK24 carrying one-copy integration of plasmid pL-CxnA_1_-F | This study |
| *Streptomyces albus*/pL-CxnB-F | *S. albus* carrying one-copy integration of plasmid pL-CxnB-F | This study |
| *Streptomyces albus*/pL-CxnA_1_-F | *S. albus* carrying one-copy integration of plasmid pL-CxnA_1_-F | This study |
| *Escherichia coli* |  |  |
| DH5α | General cloning host | [9] |
| ET12567/pUZ8002 | Donor strain for intergeneric conjugation between *E. coli* and *Streptomyces* and *A. tsinanensis*, Cm^r^, Km^r^ | [10] |
| **Plasmids** |  |  |
| pSET152 | *Streptomyces* integrative vector consisting of the φC31 integrase gene (*int*) and its attachment site (*attP*), Am^r^ | [11] |
| pL646 | pSET152 derivative containing the constitutive promoter *ermE*p*, Am^r^ | [12] |
| pL-CxnR | pL646 derivative plasmid containing 942 bp complete coding region of CxnR, Am^r^ | [5] |
| pSET-k-CxnR | A derivative of pSET152 containing the regulatory gene cxnR driven by the *kasO*p* from *S. coelicolor*, Am^r^ | This study |
| pSET-5768-CxnR | A derivative of pSET152 containing the regulatory gene cxnR driven by the *sco5768* promoter from *S. coelicolor*, Am^r^ | This study |
| pSET-g-CxnR | A derivative of pSET152 containing the regulatory gene cxnR driven by the *gapdh* promoter from *Eggerthella lenta*, Am^r^ | This study |
| pSET-r-CxnR | A derivative of pSET152 containing the regulatory gene cxnR driven by the *rpsL* promoter from *Xylanimonas cellulosilytica*, Am^r^ | This study |
| pSET-2027-CxnR | A derivative of pSET152 containing the regulatory gene cxnR driven by the *isa2027* promoter from *Streptomyces* sp. CPCC 204095, Am^r^ | This study |
| pL-CxnB-F | pL646 derivative plasmid containing CxnBCDEF, Am^r^ | [2] |
| pL-CxnA_1_-F | pL646 derivative plasmid containing CxnA_1_ABCDEF, Am^r^ | This study |

# Table S9. Primers used in this study

| **Name** | **Sequence (5'→3')** | **Purpose** |
| --- | --- | --- |
| CxnA_1_-F-1_fwd  Cxn A_1_-F-1_rev | atacagaaccactccaaggaggaccccacatATGGCAGGCGTGAAAGATG  ccggcacacgtaagtCACTTGAGCCGTTGGTGATG | Used to amplify the fragment 1 of CxnA_1_-F |
| Cxn A_1_-F-2_fwd  Cxn A_1_-F-2_rev | ccaacggctcaagtgACTTACGTGTGCCGGACG  gctcgtgaacccgacCACGTCGGGAGGATCGGC | Used to amplify the fragment 2 of CxnA_1_-F |
| Cxn A_1_-F-3_fwd  Cxn A_1_-F-3_rev | gatcctcccgacgtgGTCGGGTTCACGAGCACC  ccaagcttgggctgcaggtcgactctagagCTACTCCGTAGCACCGTC | Used to amplify the fragment 3 of CxnA_1_-F |
| ID-oriT-fw  ID-native-attB-rev | gcagagcaggattcccgttgagca  acgtcccgtgctcaccgtgacca | Used to verify the native φC31 integration site in *Streptomyces* |
| ID-CPK-attB-rev | cccatgctgtgcccgaagaa | Used to verify the artificial φC31 integration site located at the CPK BGC in *S. coelicolor*, with ID-oriT-fw |
| ID-RED-attB-rev | tccagcgcttggtggcgtacagctt | Used to verify the artificial φC31 integration site located at the RED BGC in *S. coelicolor*, with ID-oriT-fw |
| ID-CDA-attB-rev | catgacgcaacgcgaagaagagct | Used to verify the artificial φC31 integration site located at the CDA BGC in S. coelicolor, with ID-oriT-fw |
| CxnR-F  CxnR-R | cggactagtATGGAACTGGATTTGCGGC  ATTGAATTCTCAGGCCGGAGCGGTTC | Used to amplify cxnR |
| pSET152  attB_streptomyces | TTCGGCGGCTTCAAGTTCGG  CGGTGGGGGTGCCAGGG | Used to verify the φC31 integration site in *S. coelicolor* M1152 |


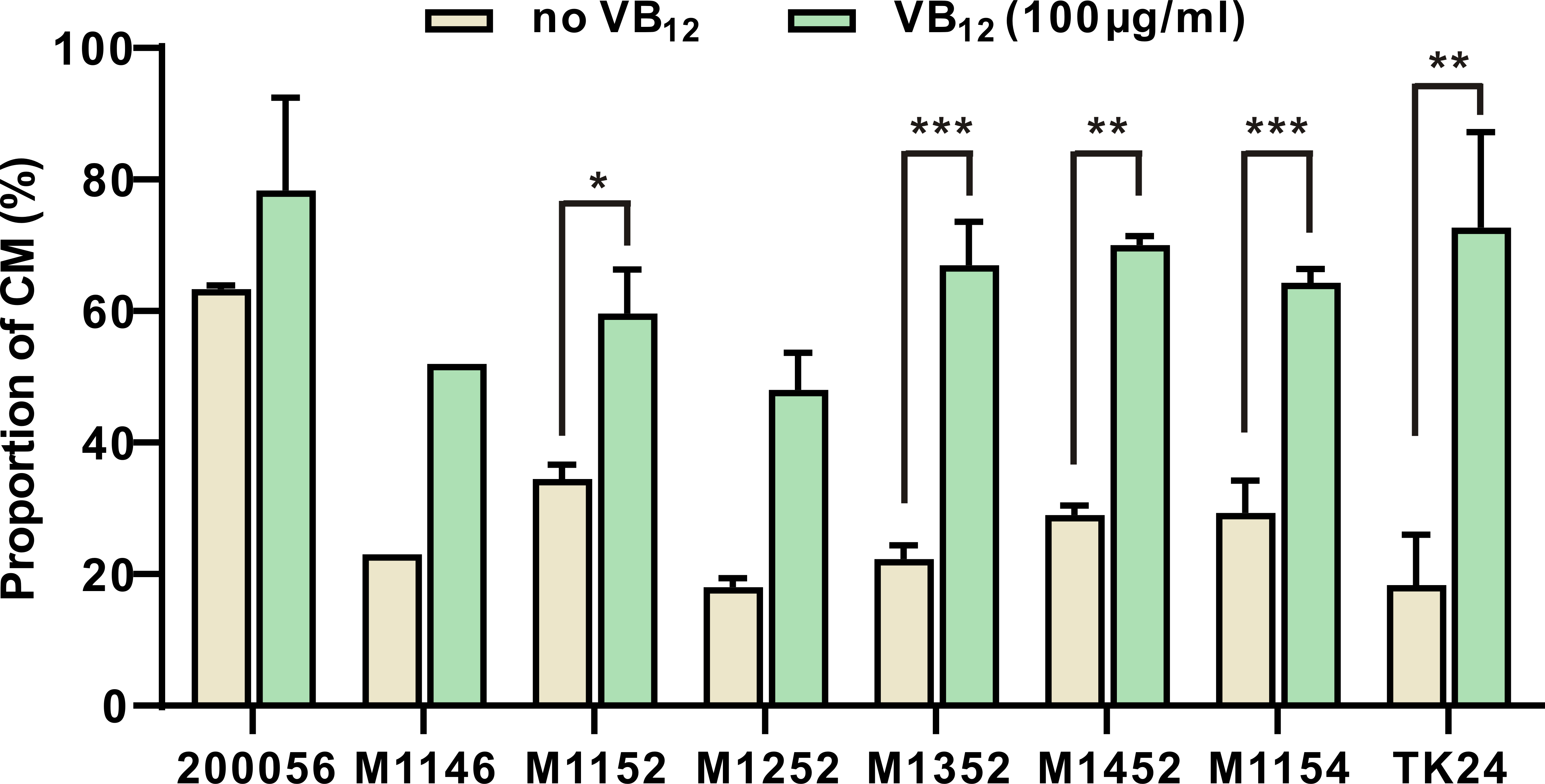


# Figure S1. The change of the proportion of CM with 100 μg/ml VB_12_ fed to ISP2 plate in *Streptomyces*/pL-CxnA_1_-F and 200056.

The proportion of CM in each strain was compared with that in VB_12_ supply (Student’s *t* test or Welch’s *t* test, ^*^*p* < 0.05, ^**^*p* < 0.01, ^***^*p* < 0.001). Values are presented as mean ± SEM (three independent conjugants for each strain).


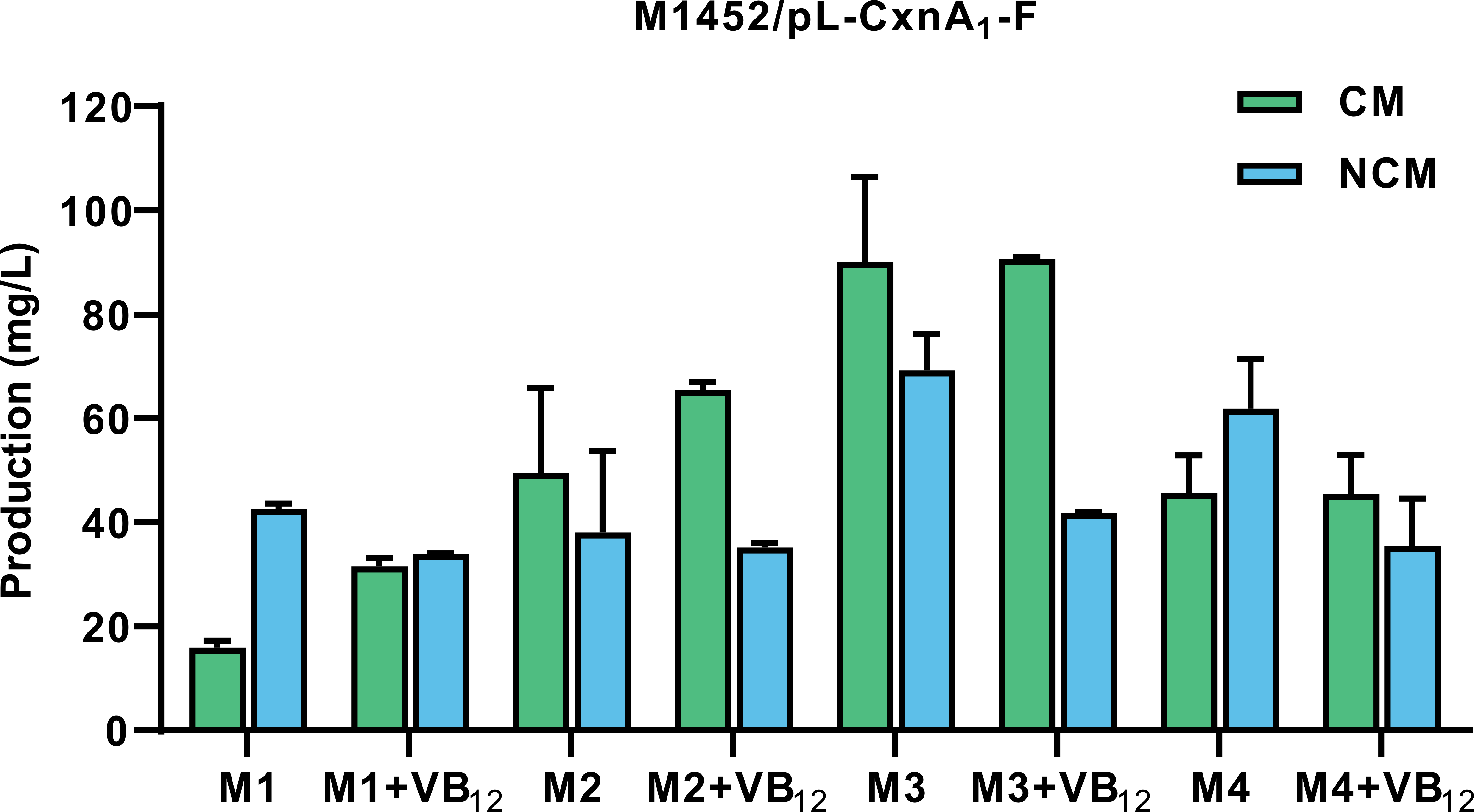


Figure S2. Production of CM and NCM in *S. coelicolor* M1452/pL-CxnA_1_-F cultivated in different fermentation media. Values are presented as mean ± SEM (three independent conjugants for each strain).


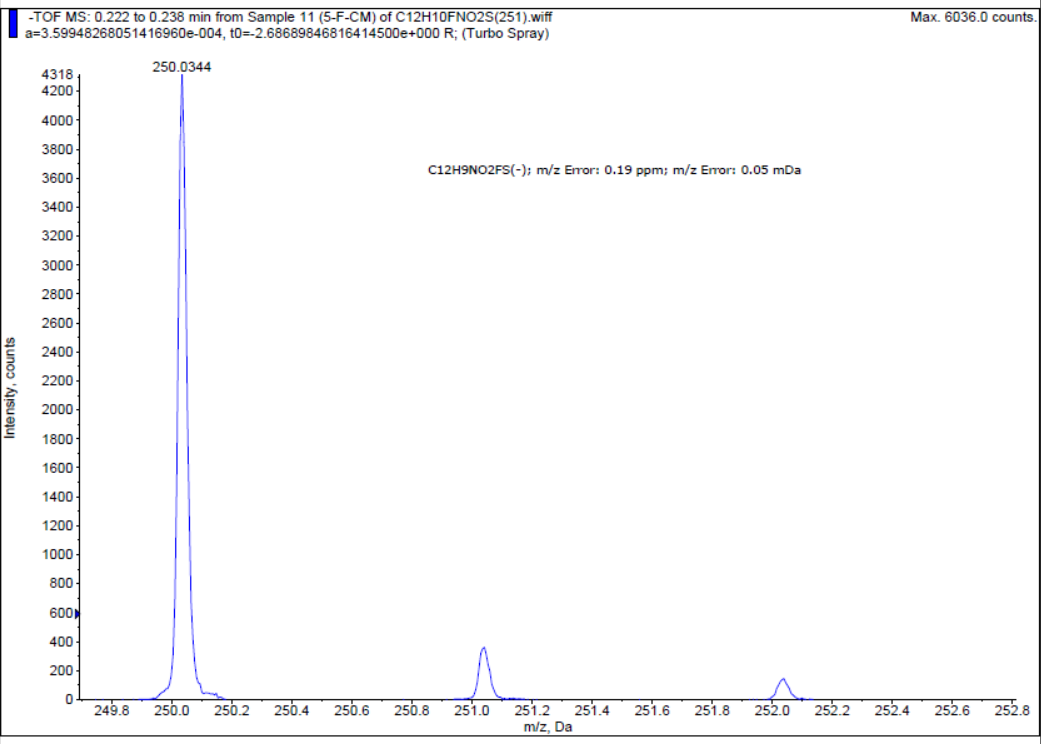


# Figure S3. (-)-HR-ESIMS spectrum of 5-F-CM


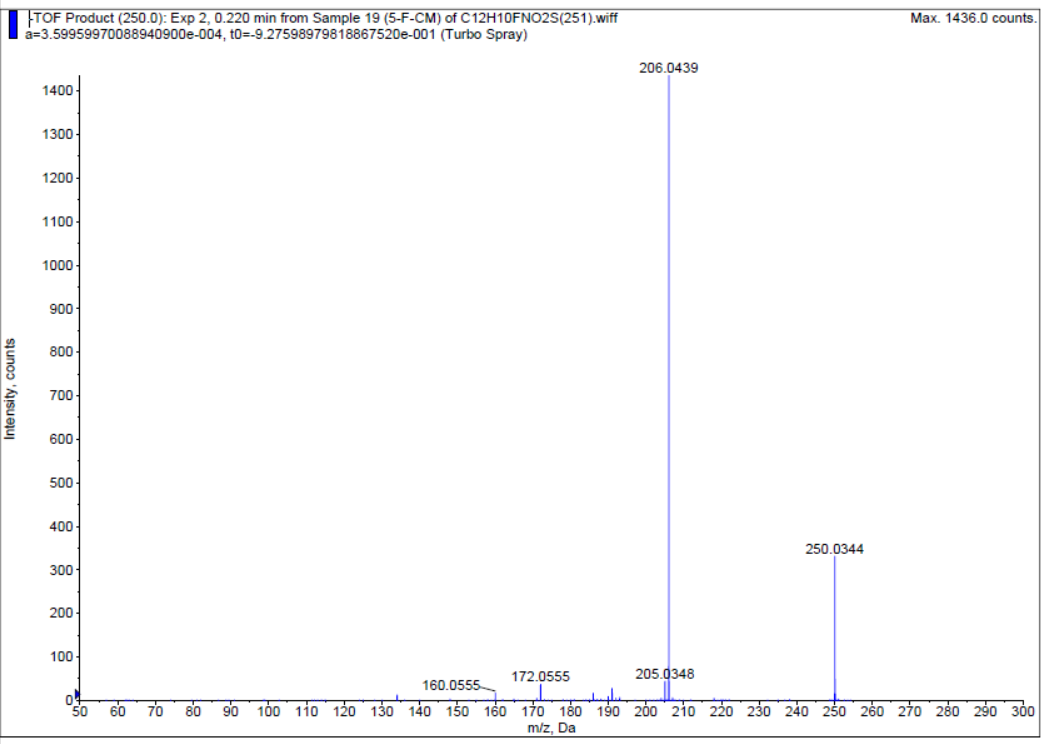

# Figure S4. (-)-HR-ESIMS/MS spectrum of 5-F-CM

# Figure S5. ^1^H NMR spectrum of 5-F-CM in CD_3_COCD_3_

# Figure S6. ^13^C NMR spectrum of 5-F-CM in CD_3_COCD_3_

# Figure S7. HSQC spectrum of 5-F-CM in CD_3_COCD_3_

# Figure S8. HMBC spectrum of 5-F-CM in CD_3_COCD_3_

# Figure S9. ^1^H-^1^H COSY spectrum of 5-F-CM in CD_3_COCD_3_


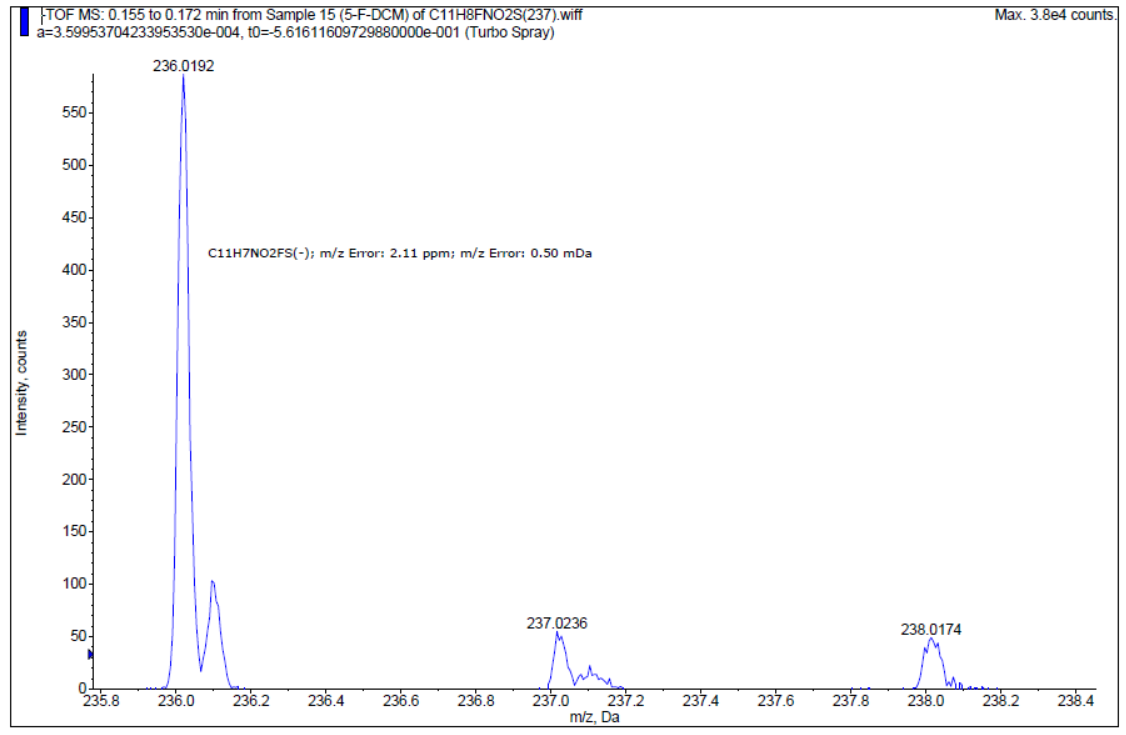


# Figure S10. (-)-HR-ESIMS spectrum of 5-F-NCM


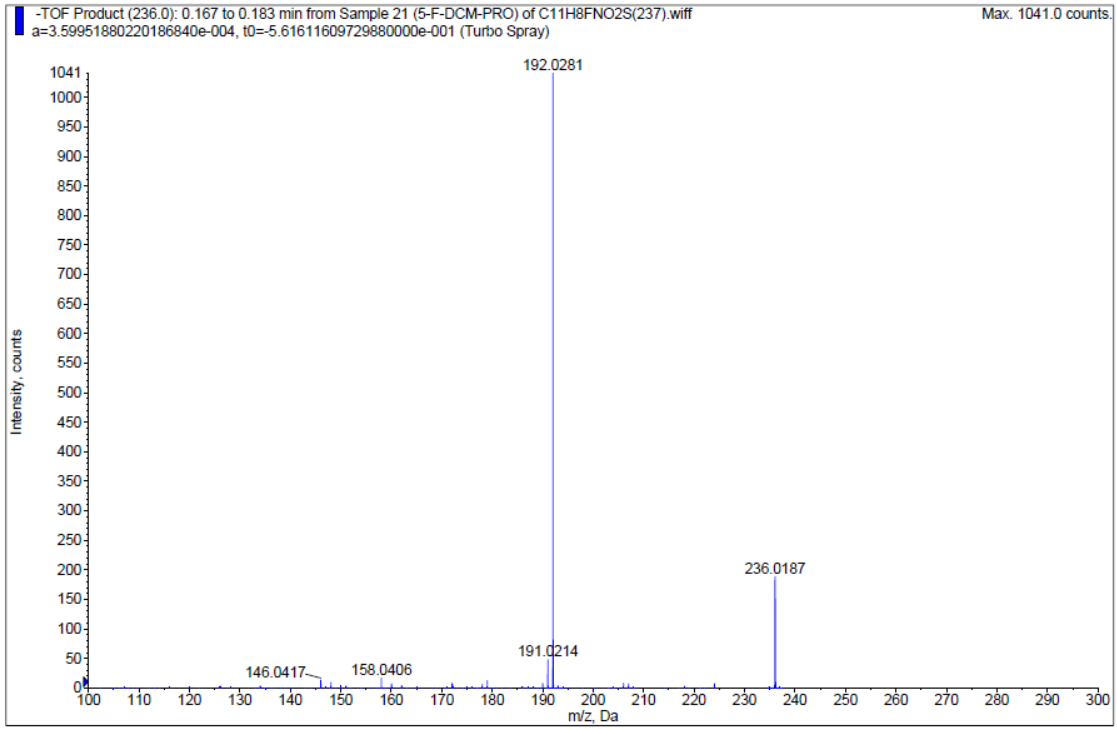

# Figure S11. (-)-HR-ESIMS/MS spectrum of 5-F-NCM

# Figure S12. ^1^H NMR spectrum of 5-F-NCM in CD_3_OD

# Figure S13. ^13^C NMR spectrum of 5-F-NCM in CD_3_OD

# Figure S14. HSQC spectrum of 5-F-NCM in CD_3_OD

# Figure S15. HMBC spectrum of 5-F-NCM in CD_3_OD

# Figure S16. ^1^H-^1^H COSY spectrum of 5-F-NCM in CD_3_OD

# Figure S17. ^1^H NMR spectrum of 7-F-NCM in CD_3_OD

# Figure S18. ^13^C NMR spectrum of 7-F-NCM in CD_3_OD

# Figure S19. HSQC spectrum of 7-F-NCM in CD_3_OD

# Figure S20. HMBC spectrum of 7-F-NCM in CD_3_OD

# Figure S21. ^1^H-^1^H COSY spectrum of 7-F-NCM in CD_3_OD


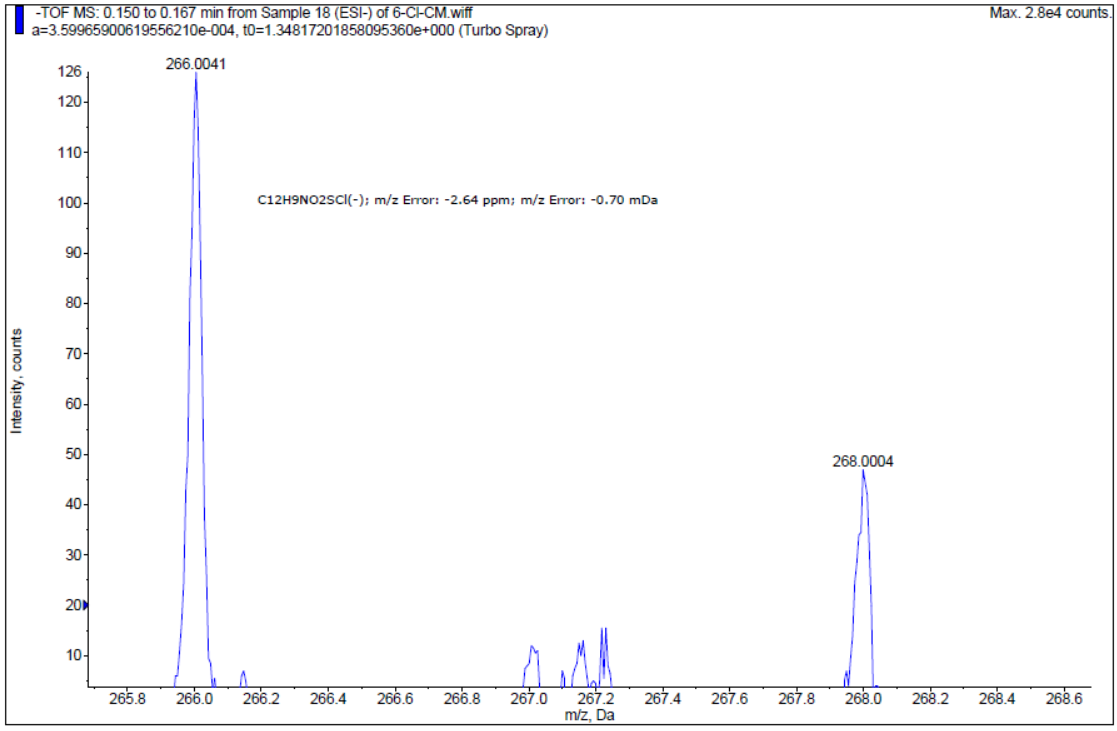


# Figure S22. (-)-HR-ESIMS spectrum of 6-Cl-CM


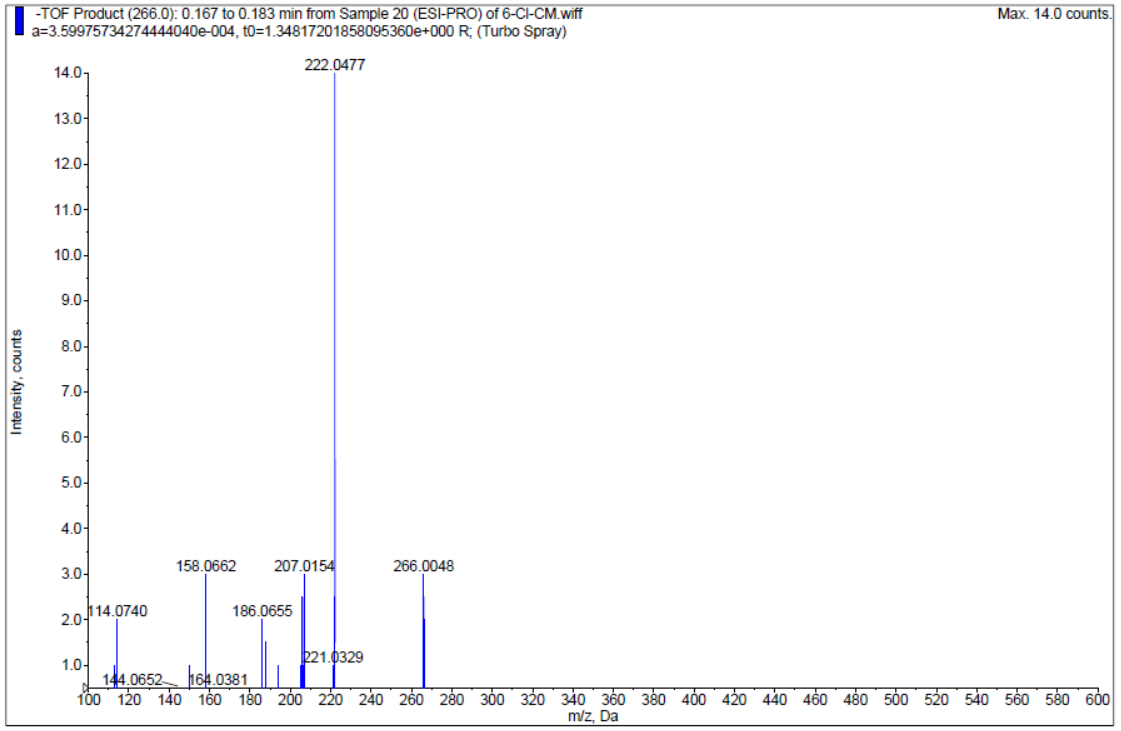

# Figure S23. (-)-HR-ESIMS/MS spectrum of 6-Cl-CM


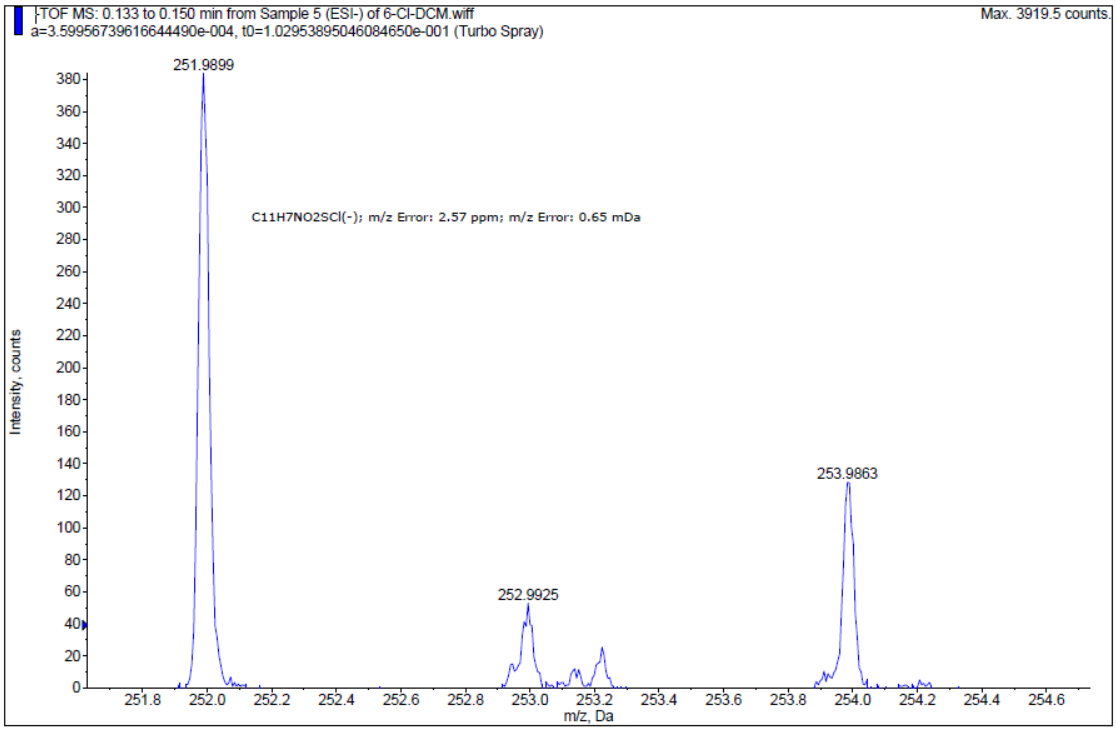


# Figure S24. (-)-HR-ESIMS spectrum of 6-Cl-NCM


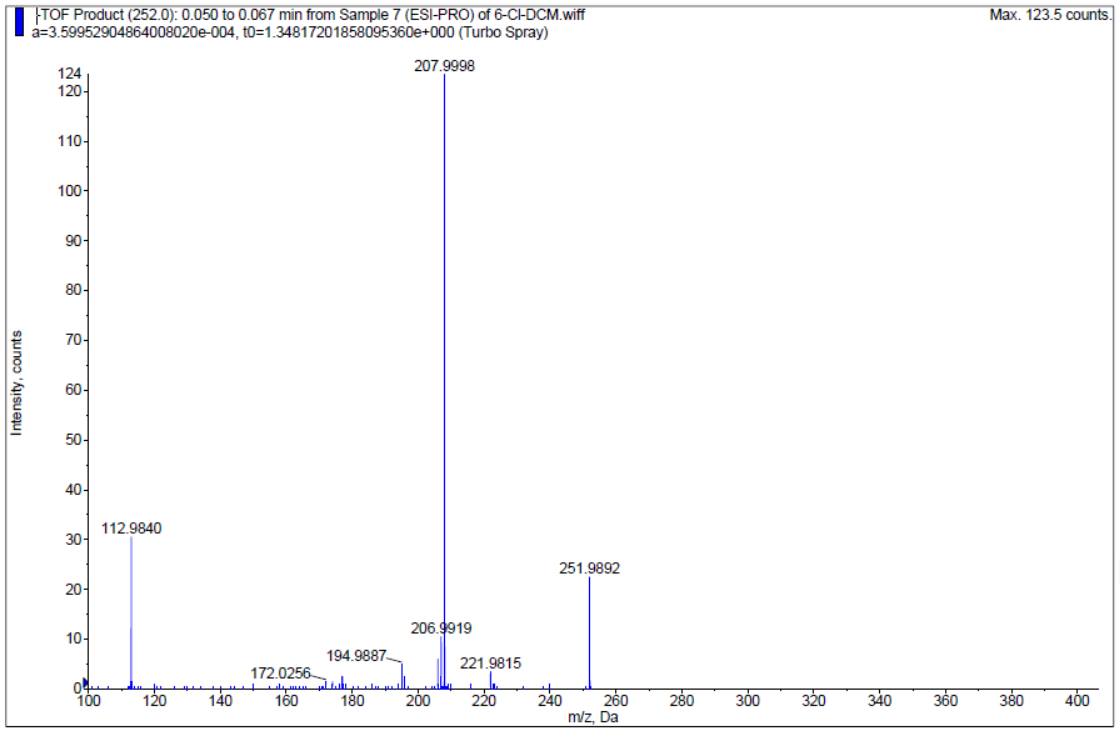

# Figure S25. (-)-HR-ESIMS/MS spectrum of 6-Cl-NCM

# Figure S26. ^1^H NMR spectrum of 6-Cl-NCM in CD_3_OD

# Figure S27. ^13^C NMR spectrum of 6-Cl-NCM in CD_3_OD

# Figure S28. HSQC spectrum of 6-Cl-NCM in CD_3_OD

# Figure S29. HMBC spectrum of 6-Cl-NCM in CD_3_OD

# Figure S30. ^1^H-^1^H COSY spectrum of 6-Cl-NCM in CD_3_OD


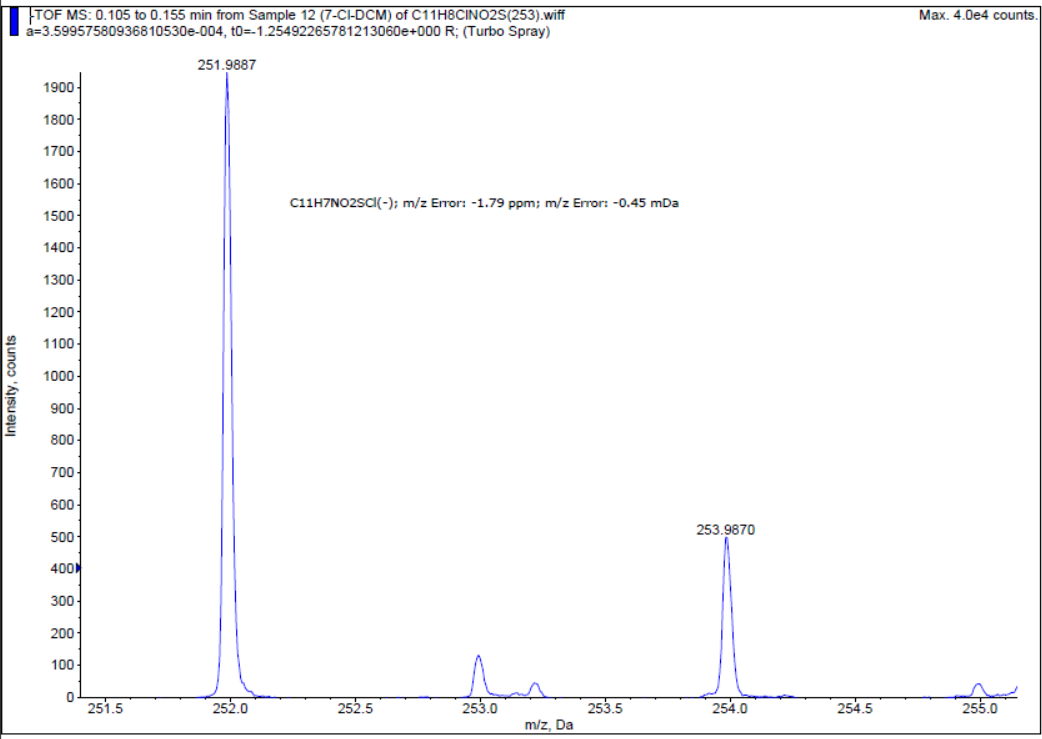


# Figure S31. (-)-HR-ESIMS spectrum of 7-Cl-NCM


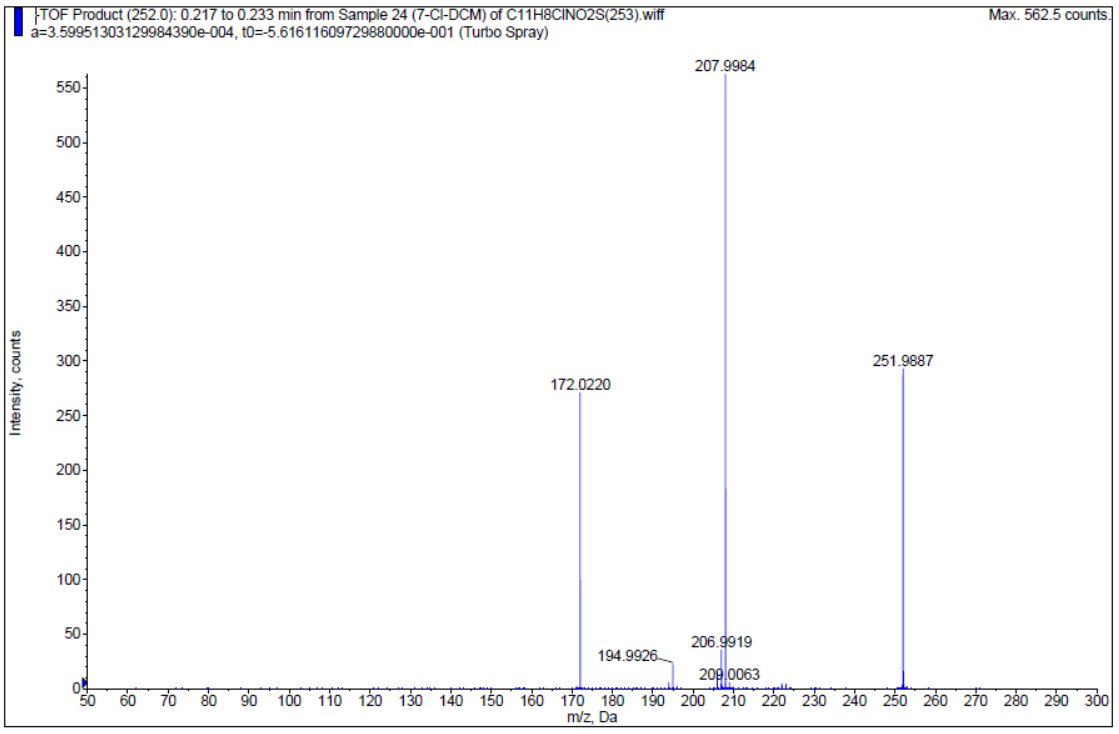

# Figure S32. (-)-HR-ESIMS/MS spectrum of 7-Cl-NCM

# Figure S33. ^1^H NMR spectrum of 7-Cl-NCM in CD_3_OD

# Figure S34. ^13^C NMR spectrum of 7-Cl-NCM in CD_3_OD

# Figure S35. HSQC spectrum of 7-Cl-NCM in CD_3_OD

# Figure S36. HMBC spectrum of 7-Cl-NCM in CD_3_OD

# Figure S37. ^1^H-^1^H COSY spectrum of 7-Cl-NCM in CD_3_OD

# References

1. Wang S., Huang J., Du Y., Huang W., Xue Y., Xu X., Zhong G., Shi Y., Hong B, Bian X., Liu W: **Cobalamin-dependent radical S-adenosyl-L-methionine protein functions with a partner to successively methylate tricyclic indole alkaloid for chuangxinmycin maturation and derivatization.** *Science China Chemistry* 2025, **68:**308-316.

2. Shi Y, Jiang Z, Hu X, Hu X, Gu R, Jiang B, Zuo L, Li X, Sun H, Zhang C, et al: **The Cytochrome P450 Catalyzing C-S Bond Formation in S-Heterocyclization of Chuangxinmycin Biosynthesis.** *Angew Chem Int Ed Engl* 2021, **60:**15399-15404.

3. Zhang X, Xu X, You C, Yang C, Guo J, Sang M, Geng C, Cheng F, Du L, Shen Y, et al: **Biosynthesis of Chuangxinmycin Featuring a Deubiquitinase-like Sulfurtransferase.** *Angew Chem Int Ed Engl* 2021, **60:**24418-24423.

4. Chuangxinmycin Research Group: **Studies on a new antibiotic-Chuangxinmycin.** *Scientia Sinica* 1977, **20:**106-112.

5. Shi Y, Jiang Z, Li X, Zuo L, Lei X, Yu L, Wu L, Jiang J, Hong B: **Biosynthesis of antibiotic chuangxinmycin from *Actinoplanes tsinanensis*.** *Acta Pharm Sin B* 2018, **8:**283-294.

6. Gomez-Escribano JP, Bibb MJ: **Engineering *Streptomyces coelicolor* for heterologous expression of secondary metabolite gene clusters.** *Microb Biotechnol* 2011, **4:**207-215.

7. Li L, Zheng G, Chen J, Ge M, Jiang W, Lu Y: **Multiplexed site-specific genome engineering for overproducing bioactive secondary metabolites in actinomycetes.** *Metab Eng* 2017, **40:**80-92.

8. Ruckert C, Albersmeier A, Busche T, Jaenicke S, Winkler A, Friethjonsson OH, Hreggviethsson GO, Lambert C, Badcock D, Bernaerts K, et al: **Complete genome sequence of *Streptomyces lividans* TK24.** *J Biotechnol* 2015, **199:**21-22.

9. Sambrook J, Russell DW: *Molecular cloning: a laboratory manual, 3rd edn.* Cold Spring Harbor: Cold Spring Harbor Laboratory 2001.

10. Paget MS, Chamberlin L, Atrih A, Foster SJ, Buttner MJ: **Evidence that the extracytoplasmic function sigma factor σ^E^ is required for normal cell wall structure in *Streptomyces coelicolor* A3(2).** *J Bacteriol* 1999, **181:**204-211.

11. Bierman M, Logan R, O'Brien K, Seno ET, Rao RN, Schoner BE: **Plasmid cloning vectors for the conjugal transfer of DNA from *Escherichia coli* to *Streptomyces* spp.** *Gene* 1992, **116:**43-49.

12. Hong B, Phornphisutthimas S, Tilley E, Baumberg S, McDowall KJ: **Streptomycin production by *Streptomyces griseus* can be modulated by a mechanism not associated with change in the *adpA* component of the A-factor cascade.** *Biotechnol Lett* 2007, **29:**57-64.
